# Qudit W and Greenberger–Horne–Zeilinger states on a two-photon chip

**DOI:** 10.1038/s41377-026-02285-7

**Published:** 2026-08-05

**Authors:** Yulin Chi, Haiyu Ding, Fei Wang, Chengkang Pan, Chunju Shao

**Affiliations:** 1 China Mobile Research Institute, China Mobile, 32 West Xuanwumen Street, Beijing, 100053 China; 2 State Key Laboratory for Mesoscopic Physics, School of Physics, Peking University, 209 Chengfu Road, Beijing, 100871 China

**Keywords:** Quantum optics, Single photons and quantum effects, Silicon photonics

## Abstract

High-dimensional quantum entangled states exhibit unique properties. While generalizations like qudit Bell, Greenberger–Horne–Zeilinger (GHZ), and Cluster states have been studied, the qudit W states remain underexplored. We discover that a superposition of qudit Dicke states can serve as high-dimensional generalizations of W states, demonstrating their enhanced robustness against qudit loss, and present linearly scalable quantum circuits for their generation. Using a programmable silicon-photonic entanglement generator, we first theoretically and experimentally demonstrate the generation of 3-ququart GHZ and W states using two photons and one ancillary ququart, with fidelities of 0.932 ± 0.009 and 0.901 ± 0.012, respectively. Genuine multipartite entanglement of the GHZ state and the improved resilience of the W state are confirmed. Our findings provide new insights and efficient approaches for qudit-based quantum science and technologies.

## Introduction

Entanglement lies at the heart of quantum technologies, endowing them with extraordinary capabilities unattainable by classical counterparts. Recent research frontiers have extended to exploring high-dimensional (qudit) entangled states^1^, which offer expanded Hilbert spaces and novel computational paradigms beyond conventional qubits. Unlike qubits, which are confined to two orthogonal bases ( ∣0〉 and  ∣1〉), qudits can be in a superposition of *d* orthogonal bases ($| 0\rangle ,| 1\rangle ,\cdots \,,| d-1\rangle$), where *d* represents the dimensionality of the quantum system. Extending quantum systems from two to higher dimensions endows them with unique properties, including the enhanced capacity and robustness of quantum communication^2–4^, more efficient computation^5–7^, hardware-efficient error correction^8–10^, and stronger quantum nonlocality^11,12^. Today, qudit entangled states have been realized in superconducting^13^, ion trap^14^, and photonic systems^15–18^, but at limited scale.

Photonic quantum systems play an irreplaceable role in the generation and distribution of entanglement, owing to their long coherence times, fast propagation speeds, and compatibility with scalable quantum architectures^19,20^. Furthermore, emerging integrated photonics technologies have provided an efficient, stable, and scalable platform for quantum applications via photonic chips^21^. These attributes are critical for enabling essential entanglement-based quantum applications, such as quantum key distribution^22^, quantum teleportation^23,24^, and the interconnection of quantum computers^25–27^, which rely on the reliable generation, transmission and detection of entangled states across distances.

Moreover, photons serve as natural carriers of high-dimensional quantum states, leveraging multiple degrees of freedom–including time-bin^4,28,29^, frequency-bin^28–30^, spatial mode^15,31^, and orbital angular momentum^18,32^–that are naturally amenable to high-dimensional encoding with minimal crosstalk. These orthogonal degrees of freedom allow for the representation of quantum states in *d*-dimensional Hilbert spaces, opening new frontiers for high-dimensional quantum information processing^1,5,33^. So far, photonic systems offer the largest-scale and most promising platform for generating qudit entanglement. Significant progress has been made in linear optical systems, which enabled the generation^15–17^ and applications^3,34–36^ of qudit Bell and GHZ states. These advancements highlight the potential of photonic platforms to harness the enhanced information capacity and nonclassical correlations inherent to qudit entanglement, paving the way for next-generation quantum technologies that surpass the limitations of binary quantum systems.

Despite these advances, challenges remain in generating qudit entangled states. The creation of GHZ states with more than four 3-dimensional photons (including 3-ququart GHZ states) by path identity^37^ without ancillary photons is prohibited^38^, and Bell measurements using linear elements and particle detectors in *d* > 3-dimensional systems without ancillary resources are impossible^39^. Schemes to address these limitations require considerable ancillary photons or resources^34,40–42^. Additionally, the definition and experimental methods for qudit W states–critical for quantum systems’ robustness to particle loss–lack clarity and a generalized framework. A further limitation is the absence of a programmable device to generate tailored qudit entangled states on demand; existing setups rely on fixed components and probabilistic schemes, limiting adaptability. These gaps–from scalability in multi-level systems to missing definitions and flexible tools–hinder progress in high-dimensional quantum information research, calling for solutions to unlock their full potential.

In this work, we propose a generalization of qudit W states as the superpositions of specific qudit Dicke states^43^, design linearly scalable and efficient quantum circuits for their generation, and present a programmable 3-ququart entanglement generator–implemented on a two-photon silicon-photonic chip with one ancillary ququart–featuring high integration, stable operation, and programmability. This device can deterministically generate various 3-ququart entangled states and perform projective measurements. Experimental results demonstrate the high-fidelity generation of the W and GHZ states, validating the device’s capability to programmably generate qudit entanglement. The stronger robustness of qudit W states against qudit loss, as predicted, has also been confirmed. This ancilla-based approach overcomes the scalability limitations of previous linear-optics-based approaches, marking a critical step toward large-scale high-dimensional quantum technologies.

## The generalized qudit W state

Many important entangled states, such as GHZ ($| {GHZ}_{n}^{d}\rangle =\frac{1}{\sqrt{d}}{\sum }_{i=0}^{d-1}{| i\rangle }^{\otimes n}$) and Cluster states, have been generalized to *d*-dimensional. Most of these generalizations are quite straightforward; however, the generalization of another commonly used entangled state–the W state–to higher dimensions is less obvious.

### Definition and properties

One existing generalization of the *d*-dimensional qudit W states is: $\frac{1}{\sqrt{(d-1)N}}{\sum }_{j=1}^{d-1}(| j0\cdots 0\rangle +| 0j\cdots 0\rangle +\cdots +| 00\cdots j\rangle )$^44^. This state can be rewritten as $\frac{1}{\sqrt{N}}(| s0\cdots 0\rangle +| 0s\cdots 0\rangle +\cdots +| 00\cdots s\rangle )$, where $| s\rangle =\frac{1}{\sqrt{d-1}}{\sum }_{j=1}^{d-1}| j\rangle$. This is equivalent to the qubit W states up to local unitary transformations. Furthermore, qubit W states are a type of Dicke states, which have been generalized to $| {D}_{n}({k}_{0},{k}_{1},\ldots ,{k}_{d-1})\rangle$^43^ – *n*-qudit equal-weight superposition states with ${k}_{0}\,| 0\rangle$’s, ${k}_{1}\,| 1\rangle$’s, … , ${k}_{d-1}\,| d-1\rangle$’s, satisfying ${\sum }_{i=0}^{d-1}{k}_{i}=n$. However, qudit Dicke states of the form $| {D}_{n}({k}_{i}=n-1,{k}_{j}=1)\rangle$ are also equivalent to qubit W states, while general qudit Dicke states $| {D}_{n}({k}_{0},{k}_{1},\ldots ,{k}_{d-1})\rangle$ are equivalent to *r*-dimensional Dicke states for *d* > *r*, where *r* ≤ *n* is the number of non-zero elements in {*k*_0_, *k*_1_, …, *k*_*d*−1_}. Thus, for *d* > *n* ≥ *r*, there must exist unused energy levels (see Supplementary Appendix A for details).

To utilize all energy levels, we re-generalize qudit W states as:

1 $$| {W}_{n}^{d}\rangle =\frac{1}{\sqrt{{C}_{d+n-2}^{n-1}}}\mathop{\sum }\limits_{{i}_{1}+{i}_{2}+\cdots +{i}_{n}=d-1}| {i}_{1},{i}_{2},\cdots \,,{i}_{n}\rangle$$

The binomial coefficient ${C}_{d+n-2}^{n-1}$ in the normalization factor denotes the number of terms satisfying *i*_1_ + *i*_2_ + ⋯ + *i*_*n*_ = *d* − 1. For example, in the case of *d* = 4, *n* = 3:

2 $$| {W}_{3}^{4}\rangle =\frac{1}{\sqrt{10}}(| 300\rangle +| 030\rangle +| 003\rangle +| 210\rangle +| 120\rangle +| 201\rangle +| 102\rangle +| 021\rangle +| 012\rangle +| 111\rangle )$$

For the state $| {W}_{n}^{d}\rangle$, we have the relation:

3 $$\begin{array}{ll}| {{\rm{W}}}_{n}^{d}\rangle = & | 0\rangle \sqrt{\frac{{C}_{d+n-3}^{n-2}}{{C}_{d+n-2}^{n-1}}}| {{\rm{W}}}_{n-1}^{d}\rangle +| 1\rangle \sqrt{\frac{{C}_{d+n-4}^{n-2}}{{C}_{d+n-2}^{n-1}}}| {{\rm{W}}}_{n-1}^{d-1}\rangle +\cdots \\ & +| d-2\rangle \sqrt{\frac{{C}_{n-1}^{n-2}}{{C}_{d+n-2}^{n-1}}}| {{\rm{W}}}_{n-1}^{2}\rangle +| d-1\rangle \sqrt{\frac{1}{{C}_{d+n-2}^{n-1}}}| {{\rm{W}}}_{n-1}^{1}\rangle \end{array}$$

In Table 1, we present the probability distributions of measurement outcomes and the resultant quantum states of the remaining *n* − 1 qudits after measuring a single qudit in the $| {W}_{n}^{d}\rangle$ state. Post-measurement, the system loses entanglement (projected onto $| {W}_{n-1}^{1}\rangle ={| 0\rangle }^{\otimes (n-1)}$) with probability $1/{C}_{d+n-2}^{n-1}$ (∝*n*^−(*d*−1)^ when *n* is sufficiently large), which is significantly lower than 1/*n* in the qubit case. This demonstrates that increasing the dimensionality enhances the robustness of qudit W states against single-qudit loss, as detailed in Supplementary Appendix B.

**Table 1:** Post-measurement state statistics after single-qudit measurement on $| {W}_{n}^{d}\rangle$

Probability	Measurement result	Post-measurement state
$1/{C}_{d+n-2}^{n-1}$	∣d-1〉	$| {W}_{n-1}^{1}\rangle$
${C}_{n-1}^{n-2}/{C}_{d+n-2}^{n-1}$	∣d-2〉	$| {W}_{n-1}^{2}\rangle$
⋮	⋮	⋮
${C}_{d+n-4}^{n-2}/{C}_{d+n-2}^{n-1}$	∣1〉	$| {W}_{n-1}^{d-1}\rangle$
${C}_{d+n-3}^{n-2}/{C}_{d+n-2}^{n-1}$	∣0〉	$| {W}_{n-1}^{d}\rangle$

It should be noted that for qudit Dicke states $| {D}_{n}({k}_{0},{k}_{1},\ldots ,{k}_{d-1})\rangle$, they satisfy our definition of qudit W states when ${\sum }_{i=0}^{d-1}i{k}_{i}=d-1$, hence qudit W states are superpositions of all such qudit Dicke states:

4 $$| {W}_{n}^{d}\rangle =\sqrt{\frac{1}{{C}_{d+n-2}^{n-1}}}\mathop{\sum }\limits_{\mathop{\sum }\limits_{i=0}^{d-1}i{k}_{i}=d-1}\sqrt{\frac{n!}{{\prod }_{j=0}^{d-1}{k}_{j}!}}| {D}_{n}({k}_{0},{k}_{1},\ldots ,{k}_{d-1})\rangle$$

For example, as illustrated in Eq. (2), we have $| {W}_{3}^{4}\rangle =\sqrt{\frac{3}{10}}| {D}_{3}(2,0,0,1)\rangle +\sqrt{\frac{6}{10}}| {D}_{3}(1,1,1,0)\rangle +\sqrt{\frac{1}{10}}| {D}_{3}(0,3,0,0)\rangle$. When *d* = 2, $| {W}_{n}^{2}\rangle =| {D}_{n}(n-1,1)\rangle$, and they both reduce to conventional *n*-qubit W states.

### Generation circuits

**Fig. 1:**
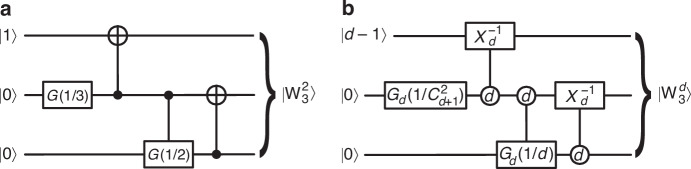
Quantum circuits for generating tripartite W states. **a** Conventional circuit for generating the 3-qubit W state $| {W}_{3}^{2}\rangle$. **b** Generalized circuit for generating the 3-qudit W state $| {W}_{3}^{d}\rangle$

Furthermore, based on the quantum circuit for generating the 3-qubit W state $| {W}_{3}^{2}\rangle$ in Fig. 1a^45^, we derive the extended circuit for generating the 3-qudit W state $| {W}_{3}^{d}\rangle$, as shown in Fig. 1b. In Supplementary Appendix C, we provide and prove a linearly scalable general quantum circuit for generating the *n*-qudit W state $| {W}_{n}^{d}\rangle$.

In Fig. 1a, the single-qubit rotation *G*(*p*) is defined as:

5 $$\begin{array}{l}G(p)=\left[\begin{array}{cc}\sqrt{p} & -\sqrt{1-p}\\ \sqrt{1-p} & \sqrt{p}\end{array}\right],\,0\le p\le 1\end{array}$$

The corresponding quantum controlled rotation is defined as *CG*(*p*) = Diag[*I*_2_, *G*(*p*)]. Accordingly, the generalized single-qudit rotation ${G}_{d}(1/{C}_{d+1}^{2})$ acting on the initial state  ∣0〉 in Fig. 1b is defined as:

6 $${G}_{d}(1/{C}_{d+1}^{2})| 0\rangle =\sqrt{\frac{1}{{C}_{d+1}^{2}}}| 0\rangle +\sqrt{\frac{{C}_{2}^{1}}{{C}_{d+1}^{2}}}| 1\rangle +\sqrt{\frac{{C}_{3}^{1}}{{C}_{d+1}^{2}}}| 2\rangle +\cdots +\sqrt{\frac{{C}_{d}^{1}}{{C}_{d+1}^{2}}}| d-1\rangle$$

In Fig. 1b, the control qudit of a 2-qudit gate is denoted by *ⓓ*. When connected to the ${X}_{d}^{-1}$ operation, the corresponding *d*-dimensional quantum controlled gate is defined as ${C}_{d}{X}_{d}^{-1}=Diag[{I}_{d},{X}_{d}^{-1},{X}_{d}^{-2},\cdots \,,{X}_{d}^{-d+1}]$, with ${X}_{d}| x\rangle =| x{\oplus }_{d}1\rangle$ and ${X}_{d}^{-1}| x\rangle =| x{\ominus }_{d}1\rangle$. ⊕ _*d*_/ ⊖ _*d*_ denote addition/subtraction modulo *d*. *I*_*d*_ represents the *d*-dimensional identity matrix. The controlled *G*_*d*_(1/*d*) operation is defined as ${C}_{d}{G}_{d}(1/d)=Diag[{I}_{d},{G}_{d}(1/2),{G}_{d}(1/3),\cdots \,,{G}_{d}(1/d)]$, where:

7 $${G}_{d}(1/j)| 0\rangle =\frac{1}{\sqrt{j}}\mathop{\sum }\limits_{i=0}^{j-1}| i\rangle ;\,j=2,3,\ldots ,d$$

We only present the *G*_*d*_ gate’s transformation for the  ∣0〉 state in Eqs. ((6) and (7)). Its full *d* × *d* matrix representation, which satisfies the unitary condition, can be chosen arbitrarily without affecting the results. *C*_*d*_*G*_*d*_(1/*d*) and Eq. (7) can be further generalized to construct the $| {W}_{n}^{d}\rangle$ state, detailed in Appendix C. We find that the quantum circuit for preparing $| {W}_{n}^{d}\rangle$ can be realized with only $2n-3={\mathcal{O}}(n)$ two-qudit controlled gates regardless of *d*. Compared with the qudit Dicke state generation circuits^43^ that scale as ${\mathcal{O}}({n}^{d})$, our circuit is much easier to implement experimentally.

Take *d* = 4 as an example, the initial state in Fig. 1b is $| 3\rangle | 0\rangle | 0\rangle$. After applying the ${G}_{4}(1/{C}_{5}^{2})$ operation on qudit 2, the quantum state of the system evolves to: $| 3\rangle (| 0\rangle +\sqrt{2}| 1\rangle +\sqrt{3}| 2\rangle +\sqrt{4}| 3\rangle )| 0\rangle /\sqrt{10}$. After the ${C}_{4}{X}_{4}^{-1}$ operation on qudit 2 (control) and 1 (target), the state becomes:

8 $$(| 3\rangle | 0\rangle +\sqrt{2}| 2\rangle | 1\rangle +\sqrt{3}| 1\rangle | 2\rangle +\sqrt{4}| 0\rangle | 3\rangle )| 0\rangle /\sqrt{10}$$

Following the application of the *C*_4_*G*_4_(1/4) gate on qudit 2 (control) and 3 (target), the quantum state evolves to:

9 $$\begin{array}{rcl} & & (| 30\rangle | 0\rangle +\sqrt{2}| 21\rangle {G}_{4}(1/2)| 0\rangle +\sqrt{3}| 12\rangle {G}_{4}(1/3)| 0\rangle +\sqrt{4}| 03\rangle {G}_{4}(1/4)| 0\rangle )/\sqrt{10}\\ & & =(| 300\rangle +| 210\rangle +| 211\rangle +| 120\rangle +| 121\rangle +| 122\rangle +| 030\rangle +| 031\rangle +| 032\rangle +| 033\rangle )/\sqrt{10}\end{array}$$

Finally, after applying the ${C}_{4}{X}_{4}^{-1}$ gate on qudits 3 (control) and 2 (target), the $| {W}_{3}^{4}\rangle$ state defined in Eq. (2) is obtained.

## Chip design and experimental setup

Our experiment relies on a two-photon four-dimensional tunable Bell-type state. We first introduce the 3-ququart entanglement-generation circuit based on this state. Subsequently, we present the corresponding silicon-photonic chip and state evolutions. Finally, we briefly introduce the chip fabrication and experimental setup.

### Entanglement generation circuits using Bell-type states

**Fig. 2:**
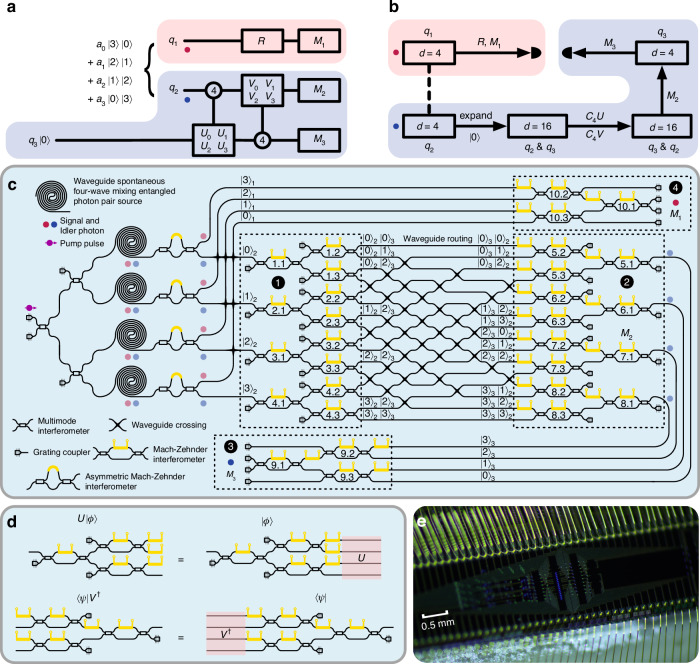
Experimental integrated photonic chip. **a** The programmable quantum circuit for generating 3-ququart W and GHZ states using Bell-type states. **b** Corresponding to (**a**) and (**c**), the on-chip Hilbert space evolution process for the generation and measurement of 3-ququart entangled states using two photons. **c** Schematic of the silicon-photonic chip in the experiment. The chip comprises a 4-dimensional Bell-type state generator, quantum state encoding/measurement structures in dashed boxes ❶–❹, and a mode routing structure between ❶ and ❷. Yellow structures are thermo-optical phase modulators for 0–2*π* phase tuning. On-chip MZIs are grouped into threes, 10 groups total. **d** The MZI group above can encode arbitrary 4-dimensional quantum state. Encoding $U| \phi \rangle$ is equivalent to first encoding $| \phi \rangle$ and then applying a 4 × 4 unitary *U*. The MZI group below can project the input quantum state onto arbitrary 4-dimensional state. Projecting onto $V| \psi \rangle$ is equivalent to first applying a 4 × 4 unitary *V*^†^ and then projecting onto $| \psi \rangle$. **e** Microscope image of the integrated photonic chip (16 mm × 1.5 mm), wire-bonded to off-chip electrical controls for programming

For experimental verification, we present programmable quantum circuits in Fig. 2a to generate 3-ququart GHZ and W states using Bell-type states. The initial states include a Bell-type two-ququart (*q*_1_, *q*_2_) entangled state ${a}_{0}| 3\rangle | 0\rangle +{a}_{1}| 2\rangle | 1\rangle +{a}_{2}| 1\rangle | 2\rangle +{a}_{3}| 0\rangle | 3\rangle$ and a third ancillary ququart (*q*_3_) initialized in  ∣0〉. We first applied a two-ququart *C*_4_*U* = Diag[*U*_0_, *U*_1_, *U*_2_, *U*_3_] gate with *q*_2_ as control and *q*_3_ as target, then a two-ququart *C*_4_*V* = Diag[*V*_0_, *V*_1_, *V*_2_, *V*_3_] gate with *q*_3_ as control and *q*_2_ as target. Finally, we performed the single-ququart gate *R* on *q*_1_.

For the W state, we set ${a}_{0}=\sqrt{\frac{1}{10}},{a}_{1}=\sqrt{\frac{2}{10}},{a}_{2}=\sqrt{\frac{3}{10}}$ and ${a}_{3}=\sqrt{\frac{4}{10}}$. The initial state in Fig. 2a becomes $\sqrt{\frac{1}{10}}| 3\rangle | 0\rangle | 0\rangle +\sqrt{\frac{2}{10}}| 2\rangle | 1\rangle | 0\rangle +\sqrt{\frac{3}{10}}| 1\rangle | 2\rangle | 0\rangle +\sqrt{\frac{4}{10}}| 0\rangle | 3\rangle | 0\rangle ,$ which is the same as Eq. (8). Thus, by setting the remaining quantum gates as *C*_4_*U* = *C*_4_*G*_4_(1/4), ${C}_{4}V={C}_{4}{X}_{4}^{-1}$ and *R* = *I*_4_, we can realize the quantum circuit in Fig. 1b for *d* = 4 and generate the $| {W}_{3}^{4}\rangle$ state defined in Eq. (2).

For the GHZ state, we set ${a}_{0}={a}_{1}={a}_{2}={a}_{3}=\frac{1}{2}$. The initial state in Fig. 2a becomes $(| 3\rangle | 0\rangle | 0\rangle +| 2\rangle | 1\rangle | 0\rangle +| 1\rangle | 2\rangle | 0\rangle +| 0\rangle | 3\rangle | 0\rangle )/2$. After setting ${C}_{4}U={C}_{4}{X}_{4}=Diag[{I}_{4},{X}_{4},{X}_{4}^{2},{X}_{4}^{3}]$ and *C*_4_*V* = Diag[*I*_4_, *I*_4_, *I*_4_, *I*_4_], the system’s quantum state before *R* becomes: $(| 3\rangle | 0\rangle | 0\rangle +| 2\rangle | 1\rangle | 1\rangle +| 1\rangle | 2\rangle | 2\rangle +| 0\rangle | 3\rangle | 3\rangle )/2$.This differs from the standard 3-ququart GHZ state only by a local unitary *R* = AntiDiag[1, 1, 1, 1] on *q*_1_. Thus, after *R*, we obtain the state $| {GHZ}_{3}^{4}\rangle =(| 0\rangle | 0\rangle | 0\rangle +| 1\rangle | 1\rangle | 1\rangle +| 2\rangle | 2\rangle | 2\rangle +| 3\rangle | 3\rangle | 3\rangle )/2$.

These quantum circuit simplifications enable practical implementation. On this basis, we designed and fabricated a programmable 3-ququart entanglement generator on a two-photon silicon-photonic chip (Fig. 2c, e) to implement Fig. 2a.

### Chip design and quantum state evolution

Figure 2c shows the chip schematic, where Mach-Zehnder interferometers (MZIs) are grouped into threes (labeled *n*. 1, *n*. 2, *n*. 3; *n* = 1, …, 10). In Fig. 2d, we present two basic on-chip MZI groups described above for state encoding and measurement. The $U| \phi \rangle$ structure expands one waveguide into four, programmably encoding any quantum state as $U| \phi \rangle$^33^. This is equivalent to first encoding an arbitrary state $| \phi \rangle$ and then performing a 4-dimensional unitary transformation *U*. Similarly, the $\langle \psi | {V}^{\dagger }$ structure compresses four waveguides into one, enabling programmable projective measurements on any quantum state $V| \psi \rangle$. This is equivalent to first applying a 4-dimensional unitary transformation *V*^†^ and then performing a projective measurement on the state $| \psi \rangle$. Therefore, we do not need to separately implement *U*_0_–*U*_3_ and *V*_0_–*V*_3_; instead, they are incorporated into MZI groups 1–4 and 5–8.

In the experiment, the 1550 nm pump laser is split evenly into four paths via on-chip multimode interferometers to pump four waveguide spontaneous four-wave mixing (SFWM) sources. The four sources, activated in superposition, convert two 1550-nm pump photons into a 1545-nm (signal, blue) and 1555-nm (idler, red) photon pair via SFWM. These pairs are separated via an asymmetric Mach-Zehnder interferometer (AMZI). Post-selecting single-photon-pair events realizes a two-photon 4-dimensional Bell state^15^: $({| 3\rangle }_{1}{| 0\rangle }_{2}+{| 2\rangle }_{1}{| 1\rangle }_{2}+{| 1\rangle }_{1}{| 2\rangle }_{2}+{| 0\rangle }_{1}{| 3\rangle }_{2})/2$. For the quantum circuit in Fig. 2a, we adjust four AMZIs to change photon-pair separation ratios and tune Bell state coefficients, yielding the programmable Bell-type state:

10 $${a}_{0}{| 3\rangle }_{1}{| 0\rangle }_{2}+{a}_{1}{| 2\rangle }_{1}{| 1\rangle }_{2}+{a}_{2}{| 1\rangle }_{1}{| 2\rangle }_{2}+{a}_{3}{| 0\rangle }_{1}{| 3\rangle }_{2}$$

where ${\sum }_{i=0}^{3}| {a}_{i}{| }^{2}=1$. Subscripts *i* here and in Fig. 2c denote *q*_*i*_ in Fig. 2a.

Next, with Eq. (10), we first introduce the overall on-chip 3-ququart entangled states’ generation and measurement process, realized via 16-dimensional encoding of one photon, two separate measurements *M*_2_, *M*_3_ on it, and *M*_1_ on the other photon. Then we analyze detailed quantum state generation for two cases: 3-ququart GHZ and W states, followed by discussions on measurement schemes and scalability.

#### Generation of the 3-ququart states with 2 photons

In Fig. 2b, we depict the evolution of on-chip two-photon Bell-type entangled state. The initial red and blue entangled photons are both 4-dimensional. For the red one encoding *q*_1_, via MZI group 10 in Fig. 2c, we first perform a single-ququart rotation *R*, followed by a 4-dimensional projective measurement *M*_1_ on *q*_1_. This corresponds to the red-background structures in Fig. 2a, b.

For the blue photon encoding *q*_2_, we expand its Hilbert space from 4-dimensional to 16-dimensional using four 4-dimensional state encoding structures (MZI groups 1–4), thereby encoding ancillary ququart *q*_3_ with an arbitrary initial state (here set to  ∣0〉). Then we implement programmable *C*_4_*U* and *C*_4_*V* gates via two sets of four 4-dimensional unitary transformations (*U*_0_–*U*_3_ encoded in MZI groups 1–4; *V*_0_–*V*_3_ in MZI groups 5–8) separated by waveguide routing (swap *q*_2_ and *q*_3_). After that, four identical 4-dimensional projective measurements *M*_2_ (MZI groups 5–8) project the 16-dimensional space onto a 4-dimensional subspace, enabling selective measurement of *q*_2_ while retaining *q*_3_. Finally, *q*_3_ is measured by *M*_3_ (MZI groups 9). This corresponds to the blue-background structures in Fig. 2a, b.

#### Generation of the 3-ququart GHZ state

For the GHZ state, we set ${a}_{0}={a}_{1}={a}_{2}={a}_{3}=\frac{1}{2}$ in Eq. (10). MZI groups 1–4 in dashed box ❶ expand the quantum state of *q*_2_ from 4 to 16 dimensions. Each group can programmably prepare any four-dimensional quantum states set as ${U}_{0}{| 0\rangle }_{3}$, ${U}_{1}{| 0\rangle }_{3}$, ${U}_{2}{| 0\rangle }_{3}$, and ${U}_{3}{| 0\rangle }_{3}$ (as illustrated in Fig. 2d). This can be written as:

11 $$\begin{array}{ll}{| 0\rangle }_{2}\to {| 0\rangle }_{2}{U}_{0}{| 0\rangle }_{3}={| 0\rangle }_{2}{| 0\rangle }_{3} & {| 1\rangle }_{2}\to {| 1\rangle }_{2}{U}_{1}{| 0\rangle }_{3}={| 1\rangle }_{2}{| 1\rangle }_{3}\\ {| 2\rangle }_{2}\to {| 2\rangle }_{2}{U}_{2}{| 0\rangle }_{3}={| 2\rangle }_{2}{| 2\rangle }_{3} & {| 3\rangle }_{2}\to {| 3\rangle }_{2}{U}_{3}{| 0\rangle }_{3}={| 3\rangle }_{2}{| 3\rangle }_{3}\end{array}$$

where we set *U*_0_ = *I*, *U*_1_ = *X*_4_, ${U}_{2}={X}_{4}^{2}$, and ${U}_{3}={X}_{4}^{3}$. Thus, the quantum state of the system is obtained as $({| 3\rangle }_{1}{| 0\rangle }_{2}{| 0\rangle }_{3}+{| 2\rangle }_{1}{| 1\rangle }_{2}{| 1\rangle }_{3}+{| 1\rangle }_{1}{| 2\rangle }_{2}{| 2\rangle }_{3}+{| 0\rangle }_{1}{| 3\rangle }_{2}{| 3\rangle }_{3})/2$. (This realizes the *C*_4_*U* = *C*_4_*X*_4_ gate with *q*_2_ as control and *q*_3_ initiated in ${| 0\rangle }_{3}$ as target.) After a single-ququart gate *R* = AntiDiag[1, 1, 1, 1] (encoded in MZI group 10) on *q*_1_, it becomes:

12 $$| {\mathrm{GHZ}}_{3}^{4}\rangle =({| 0\rangle }_{1}{| 0\rangle }_{2}{| 0\rangle }_{3}+{| 1\rangle }_{1}{| 1\rangle }_{2}{| 1\rangle }_{3}+{| 2\rangle }_{1}{| 2\rangle }_{2}{| 2\rangle }_{3}+{| 3\rangle }_{1}{| 3\rangle }_{2}{| 3\rangle }_{3})/2$$

thereby realizing the 3-ququart GHZ state. After the dashed box ❶, a waveguide-routing mesh reorders the quantum states from ${| i\rangle }_{2}{| j\rangle }_{3}$ to ${| j\rangle }_{3}{| i\rangle }_{2}$ (*i*, *j* = 0, 1, 2, 3). This realizes a swap operation between *q*_2_ and *q*_3_. For the GHZ state, it retains its original form, and the gates *V*_0_, *V*_1_, *V*_2_, *V*_3_ encoded in the dashed box ❷ (MZI groups 5–8) are set to identity.

#### Generation of the 3-ququart W state

For the W state, we set ${a}_{0}=\sqrt{\frac{1}{10}}$, ${a}_{1}=\sqrt{\frac{2}{10}}$, ${a}_{2}=\sqrt{\frac{3}{10}}$ and ${a}_{3}=\sqrt{\frac{4}{10}}$ in Eq. (10). Then, the MZI groups 1–4 in dashed box ❶ perform the expansions:

13 $$\begin{array}{rcl}{| 0\rangle }_{2}\to {| 0\rangle }_{2}{U}_{0}{| 0\rangle }_{3} & = & {| 0\rangle }_{2}{| 0\rangle }_{3}\\ {| 1\rangle }_{2}\to {| 1\rangle }_{2}{U}_{1}{| 0\rangle }_{3} & = & {| 1\rangle }_{2}({| 0\rangle }_{3}+{| 1\rangle }_{3})/\sqrt{2}\\ {| 2\rangle }_{2}\to {| 2\rangle }_{2}{U}_{2}{| 0\rangle }_{3} & = & {| 2\rangle }_{2}({| 0\rangle }_{3}+{| 1\rangle }_{3}+{| 2\rangle }_{3})/\sqrt{3}\\ {| 3\rangle }_{2}\to {| 3\rangle }_{2}{U}_{3}{| 0\rangle }_{3} & = & {| 3\rangle }_{2}({| 0\rangle }_{3}+{| 1\rangle }_{3}+{| 2\rangle }_{3}+{| 3\rangle }_{3})/\sqrt{4}\end{array}$$

where we set *U*_0_ = *I*_*d*_, *U*_1_ = *G*_4_(1/2), *U*_2_ = *G*_4_(1/3), and *U*_3_ = *G*_4_(1/4) according to Eq. (7) (to realize the *C*_4_*U* = *C*_4_*G*_4_(1/4) gate with *q*_2_ as control and *q*_3_ initiated in ${| 0\rangle }_{3}$ as target). Therefore, we obtain the quantum state:

14 $$\begin{array}{rcl} & & ({| 3\rangle }_{1}{| 0\rangle }_{2}{| 0\rangle }_{3}+{| 2\rangle }_{1}{| 1\rangle }_{2}{| 0\rangle }_{3}+{| 2\rangle }_{1}{| 1\rangle }_{2}{| 1\rangle }_{3}+{| 1\rangle }_{1}{| 2\rangle }_{2}{| 0\rangle }_{3}+{| 1\rangle }_{1}{| 2\rangle }_{2}{| 1\rangle }_{3}\\ & & +{| 1\rangle }_{1}{| 2\rangle }_{2}{| 2\rangle }_{3}+{| 0\rangle }_{1}{| 3\rangle }_{2}{| 0\rangle }_{3}+{| 0\rangle }_{1}{| 3\rangle }_{2}{| 1\rangle }_{3}+{| 0\rangle }_{1}{| 3\rangle }_{2}{| 2\rangle }_{3}+{| 0\rangle }_{1}{| 3\rangle }_{2}{| 3\rangle }_{3})/\sqrt{10}\end{array}$$

After the waveguide-routing mesh to swap *q*_2_ & *q*_3_, the quantum state is reordered to:

15 $$\begin{array}{rcl} & & ({| 3\rangle }_{1}{| 0\rangle }_{3}{| 0\rangle }_{2}+{| 2\rangle }_{1}{| 0\rangle }_{3}{| 1\rangle }_{2}+{| 2\rangle }_{1}{| 1\rangle }_{3}{| 1\rangle }_{2}+{| 1\rangle }_{1}{| 0\rangle }_{3}{| 2\rangle }_{2}+{| 1\rangle }_{1}{| 1\rangle }_{3}{| 2\rangle }_{2}\\ & & +{| 1\rangle }_{1}{| 2\rangle }_{3}{| 2\rangle }_{2}+{| 0\rangle }_{1}{| 0\rangle }_{3}{| 3\rangle }_{2}+{| 0\rangle }_{1}{| 1\rangle }_{3}{| 3\rangle }_{2}+{| 0\rangle }_{1}{| 2\rangle }_{3}{| 3\rangle }_{2}+{| 0\rangle }_{1}{| 3\rangle }_{3}{| 3\rangle }_{2})/\sqrt{10}\end{array}$$

After that, MZI groups 5–8 in dashed box ❷ are programmed to implement four 4-dimensional unitary operations on *q*_2_: *V*_0_ = *I*_4_ (for *q*_3_ in ${| 0\rangle }_{3}$), ${V}_{1}={X}_{4}^{-1}$ (for *q*_3_ in ${| 1\rangle }_{3}$), ${V}_{2}={X}_{4}^{-2}$ (for *q*_3_ in ${| 2\rangle }_{3}$), ${V}_{3}={X}_{4}^{-3}$ (for *q*_3_ in ${| 3\rangle }_{3}$) respectively. (This realizes quantum controlled gate ${C}_{4}V={C}_{4}{X}_{4}^{-1}$ with *q*_3_ as control and *q*_2_ as target.) The resulting quantum state is:

16 $$\begin{array}{rcl} & & ({| 3\rangle }_{1}{| 0\rangle }_{3}{| 0\rangle }_{2}+{| 2\rangle }_{1}{| 0\rangle }_{3}{| 1\rangle }_{2}+{| 2\rangle }_{1}{| 1\rangle }_{3}{| {\bf{0}}\rangle }_{2}+{| 1\rangle }_{1}{| 0\rangle }_{3}{| 2\rangle }_{2}+{| 1\rangle }_{1}{| 1\rangle }_{3}{| {\bf{1}}\rangle }_{2}\\ & & +{| 1\rangle }_{1}{| 2\rangle }_{3}{| {\bf{0}}\rangle }_{2}+{| 0\rangle }_{1}{| 0\rangle }_{3}{| 3\rangle }_{2}+{| 0\rangle }_{1}{| 1\rangle }_{3}{| {\bf{2}}\rangle }_{2}+{| 0\rangle }_{1}{| 2\rangle }_{3}{| {\bf{1}}\rangle }_{2}+{| 0\rangle }_{1}{| 3\rangle }_{3}{| {\bf{0}}\rangle }_{2})/\sqrt{10}\end{array}$$

Bold denotes post-operation changes. Thereafter, we get the 4-ququart W state $| {W}_{3}^{4}\rangle$ defined in Eq. (2). The single-ququart gate *R* encoded in MZI group 10 is set to identity.

#### Measurement of the generated 3-ququart states

To measure the generated 3-ququart entanglement, MZI groups 5–8 in the dashed box ❷ project the quantum state consisting of *q*_3_ *&* *q*_2_ from 16-dimensional to 4-dimensional space. By setting the projective measurements of MZI groups 5–8 as *M*_2_*V*_0_, *M*_2_*V*_1_, *M*_2_*V*_2_, and *M*_2_*V*_3_, respectively, we first implement the programmable *C*_4_*V* gate and then perform the corresponding projective measurement *M*_2_ on *q*_2_ (consistent with Fig. 2d). This enables the measurement of *q*_2_ while retaining the post-measurement state of *q*_3_ for subsequent operations. After that, MZI groups 9 and 10 in the dashed boxes ❸ and ❹ perform measurements *M*_3_ on *q*_3_ and *M*_1_ on *q*_1_, respectively. Thus, the quantum circuits in Fig. 2a can be implemented to realize the programmable generation and measurement of 3-ququart entangled states.

#### Scaling the entanglement generator

Usually, schemes employing high-dimensional encoding for *n* qudits suffer from scalability issues, as the required Hilbert space dimension scales as *d*^*n*^. Herein, by leveraging the separable properties of *M*_2_ and *M*_3_, we propose a novel scheme where the required Hilbert space dimension does not exceed *d*^2^ regardless of the value of *n*. As an example, by cascading the structure from ❶ to ❷ in Fig. 2c, we can add more ancillary ququarts and build arbitrary-size 4-dimensional programmable generators of linearly entangled states (including GHZ, Cluster, and the proposed W states), with resources scaling linearly with the qudit number. Details are in Supplementary Appendix D. With linearly scalable preparation circuits, this linear hardware scalability offers a new ancilla-based scheme for large-scale generation and projective measurement of these states.

### Chip fabrication and experimental setup

The silicon-photonic chip is fabricated on an 8-inch silicon-on-insulator wafer via standard complementary metal-oxide-semiconductor (CMOS) processes (detailed in Supplementary Appendix E), consisting of 205 optical components. During the experiment, a 150 mW 1550-nm continuous-wave laser (the pump) is coupled from a single-mode fiber to the integrated photonic chip via a grating coupler (GC). The on-chip-generated 1545-nm signal and 1555-nm idler photons are coupled into two single-mode fibers through two GCs, respectively. After filtering out the pump light, the photons are detected via superconducting nanowire single-photon detectors (SNSPDs). Two-photon coincidence counts (up to ~kHz during experiments) are selected as the photonic signals. More details of the experimental process are in Supplementary Appendix F.

## Results

We define the fidelity of the density matrix as $F=Tr[\rho {\rho }_{0}]$, where *ρ* is the density matrix reconstructed from measurements, and *ρ*_0_ is the density matrix of the target (pure) state. This definition of density matrix fidelity enables the fidelity of GHZ or W states to be determined by measuring only the non-zero elements in the real part of the target state’s density matrix (details in Supplementary Appendix G). The quantum state distribution fidelity is defined as ${F}_{s}={({\sum }_{i}\sqrt{{p}_{i}{q}_{i}})}^{2}$, where *p*_*i*_ and *q*_*i*_ denote the measured and ideal distribution probabilities, respectively. The average absolute error is defined as $\overline{| \Delta | }={\sum }_{i=0}^{N}| \langle {E}_{i}\rangle -{E}_{i}^{(0)}| /N$ where 〈*E*_*i*_〉 and ${E}_{i}^{(0)}$ denote the measured and ideal expectations, respectively.

In higher dimensions, Pauli matrices generalize to Gell-Mann matrices (GMMs). For four dimensions, there are 15 GMMs (excluding the identity), organized into three groups: six symmetric matrices (${G}_{s}^{ij}$), six antisymmetric matrices (${G}_{a}^{ij}$), and three diagonal matrices (${G}_{4}^{1}$, ${G}_{4}^{2}$ and ${G}_{4}^{3}$), with *i*, *j* ∈ {0, 1, 2, 3} and *i* < *j*. Their detailed matrix representations are provided in Supplementary Appendix G.

### Results for the 3-ququart GHZ state

For the 3-ququart GHZ state, we define 6 coherence operators ${\Omega }_{\theta }(i,j)=\cos \theta {G}_{s}^{ij}+\sin \theta {G}_{a}^{ij}$, where *i*, *j* ∈ {0, 1, 2, 3}; *i* < *j*. The corresponding eignstates and eignvalues are $(| i\rangle \pm {e}^{i\theta }| j\rangle )/\sqrt{2}$ with eignvalues ±1 and $| k\ne i,j\rangle$ with eignvalue 0. The expectation $\langle {\Omega }_{\theta }^{\otimes 3}(i,j)\rangle$ satisfies the relation:

17 $$\langle {\Omega }_{0}^{\otimes 3}(i,j)\rangle -\langle {\Omega }_{\pi /3}^{\otimes 3}(i,j)\rangle +\langle {\Omega }_{2\pi /3}^{\otimes 3}(i,j)\rangle =6{\mathrm{Re}}[\langle iii| \rho | jjj\rangle ]$$

Thus we can measure the expectations $\langle {\Omega }_{0}^{\otimes 3}(i,j)\rangle$, $\langle {\Omega }_{\pi /3}^{\otimes 3}(i,j)\rangle$ and $\langle {\Omega }_{2\pi /3}^{\otimes 3}(i,j)\rangle$ to calculate the real part of the off-diagonal density matrix elements $Re[\langle iii| \rho | jjj\rangle ]$. Additionally, by measuring the probability distribution of the quantum state in the 4^3^ = 64 computational bases  ∣ijk〉 (*i*, *j*, *k* ∈ {0, 1, 2, 3}), we can obtain all diagonal elements $\langle ijk| \rho | ijk\rangle$ of *ρ*.

**Fig. 3:**
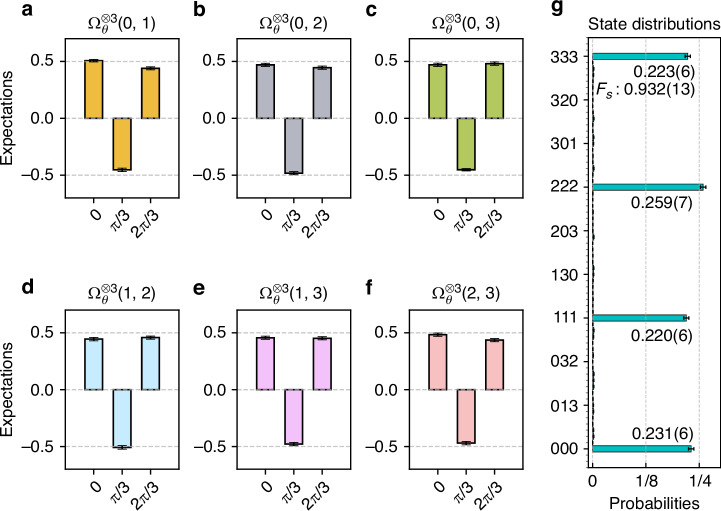
Characterization results of the 3-ququart GHZ state. **a**–**f** Results of the expectations $\langle {\Omega }_{\theta }^{\otimes 3}(i,j)\rangle$ on the generated state where *i*, *j* ∈ {0, 1, 2, 3}, *i* < *j* and *θ* ∈ {0, *π*/3, 2*π*/3}. The average absolute error from the ideal values (±0.5) is $\overline{| \Delta | }=0.036\pm 0.014$. **g** Results of the probability distribution of the quantum states in the computational basis  ∣i,j,k〉 where *i*, *j*, *k* ∈ {0, 1, 2, 3}. Errors are estimated from the photon Poisson statistics

Based on the programmable silicon-photonic chip in Fig. 2, we generated and measured the 3-ququart GHZ state. The corresponding results of six sets of ${\Omega }_{\theta }^{\otimes 3}(i,j)$, with each set corresponding to *θ* = 0, *π*/3, 2*π*/3, are shown in Fig. 3a–f with an average absolute error $\overline{| \Delta | }=0.036\pm 0.014$. The measurement results in the computational basis are shown in Fig. 3g, where the quantum state distribution fidelity is measured to be *F*_*s*_ = 0.932 ± 0.013.

**Fig. 4:**
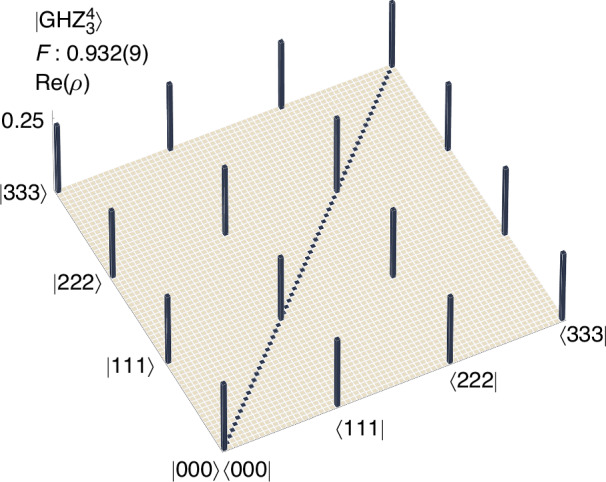
The reconstructed partial density matrix of the $| {GHZ}_{3}^{4}\rangle$ state. Navy represents the real parts of the measured diagonal elements and non-zero off-diagonal elements of the density matrix, while beige indicates the unmeasured elements

Using the data presented in Fig. 3, we reconstructed the real part of the partial density matrix of the 3-ququart GHZ state in Fig. 4. Leveraging these data, we calculated the fidelity of the quantum state to be *F* = 0.932 ± 0.009. For a general qudit $| {GHZ}_{n}^{d}\rangle$ state, if the fidelity *F* satisfies *F* > (*d* − 1)/*d*, the quantum state is genuinely entangled at least in the dimension of *d*^46^. For *d* = 4, it becomes *F* > 0.75, which is satisfied in our high-fidelity results by at least 20*σ*.

### Results for the 3-ququart W state

For the 3-ququart W state defined in Eq. (2), its density matrix contains 10 diagonal elements and 45 upper off-diagonal elements. We define 45 coherence operators to characterize them. For a general element $\langle abc| \rho | def\rangle$ where *a*, *b*, *c*, *d*, *e*, *f* ∈ {0, 1, 2, 3} and *a* + *b* + *c* = *d* + *e* + *f* = 3, we define ${\Omega }_{\theta }^{abc-def}={\Omega }_{\theta }(a,d)\otimes {\Omega }_{\theta }(b,e)\otimes {\Omega }_{\theta }(c,f)$, with:

18 $${\Omega }_{\theta }(x,y)=\left\{\begin{array}{l}\cos \theta {G}_{s}^{xy}+\sin \theta {G}_{a}^{xy}\,\,\,\,\,\,\,\,\,x < y\\ {\Omega }_{-\theta }(y,x)\qquad\qquad\qquad x > y\\ | x\rangle \langle x|\qquad\qquad\qquad\quad\,\,\, x=y\end{array}\right.$$

The corresponding real part of the densiy matrix element $\langle abc| \rho | def\rangle$ can be calculated with:

19 $${\rm{R}}{\rm{e}}[\langle abc| \rho | def\rangle ]=\left\{\begin{array}{l}\frac{1}{6}({\Omega }_{0}^{abc-def}-{\Omega }_{\pi /3}^{abc-def}+{\Omega }_{2\pi /3}^{abc-def})\,\,\,\,\,a\ne d,b\ne e,c\ne f\\ \frac{1}{4}({\Omega }_{0}^{abc-def}-{\Omega }_{\pi /2}^{abc-def})\,\,\,\,\,\,\,\,\,\,\,\,\,\,\,\,\,\,\,\,\,\,\,\,\,\,\,\,\,\,\,{\mathrm{else}}\end{array}\right.$$

A detailed list of 45 coherence operators ${\Omega }_{\theta }^{abc-def}$ are provided in Supplementary Appendix G, along with mathematical derivations and proofs.

**Fig. 5:**
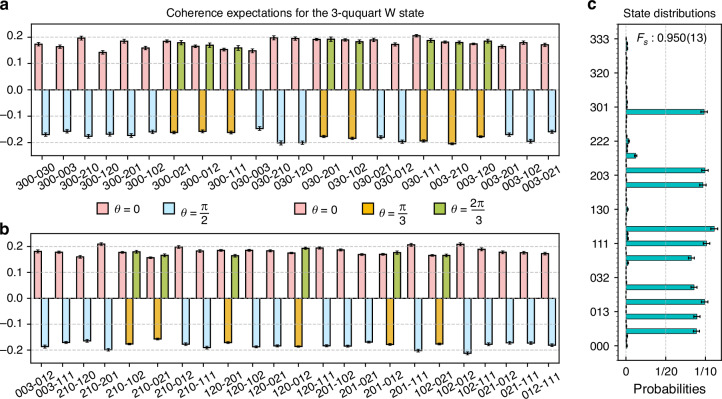
Characterization results of the 3-ququart W state. **a**, **b** Results of the 45 $\langle {\Omega }_{\theta }^{abc-def}\rangle$ expectations (denoted as *abc* − *def* on the x-axis) for the generated state. The average absolute error from the ideal values (±0.2) is $\overline{| \Delta | }=0.022\pm 0.006$. **c** Probability distribution in the computational basis  ∣i,j,k〉 with *i*, *j*, *k* ∈ {0, 1, 2, 3} for the quantum state. Errors are estimated from photon Poisson statistics

In Fig. 5a, b, we show the measured expectations of the 45 coherence operators. These correspond to the off-diagonal elements in the density matrix of the on-chip generated 3-ququart W state. For ${\Omega }_{\theta }^{abc-def}$, when *a* ≠ *d*, *b* ≠ *e*, and *c* ≠ *f*, there are three expectations corresponding to *θ* = 0, *π*/3, and 2*π*/3, which are represented by red, yellow, and green bars, respectively. In other cases, there are two expectations corresponding to *θ* = 0 and *π*/2, represented by red and blue bars, respectively. The average absolute error is measured to be $\overline{| \Delta | }=0.022\pm 0.006$. We also measured the probability distribution of the quantum state in the computational basis, as shown in Fig. 5c. The distribution fidelity is measured to be *F*_*s*_ = 0.950 ± 0.013.

**Fig. 6:**
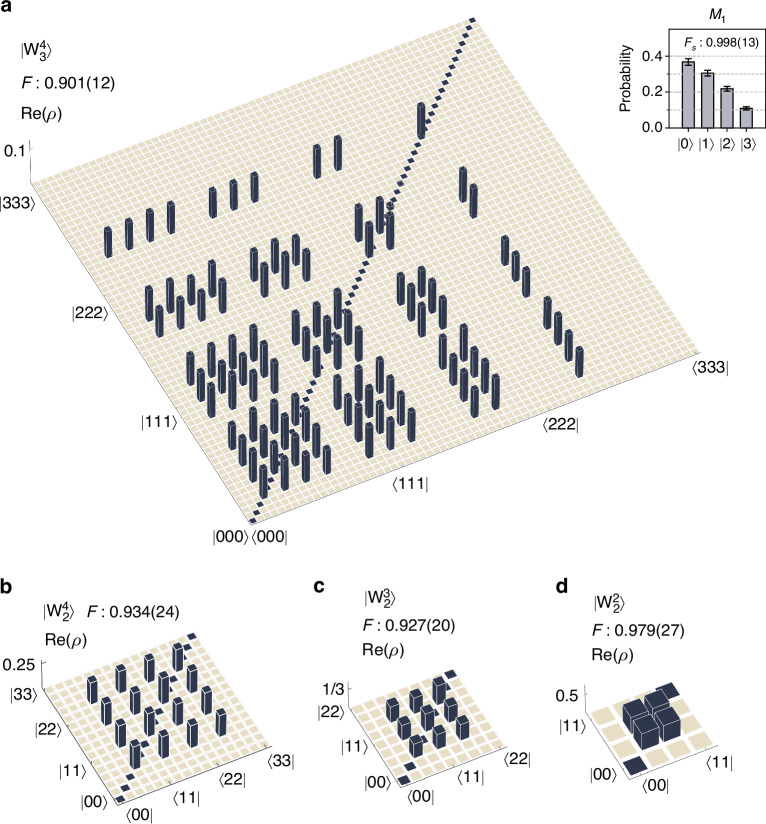
The reconstructed partial density matrices of the ${{|}{\boldsymbol{W}}_{\bf{3}}^{\bf{4}}{\rangle}}$ state and subsystems after projective measurement on ${\boldsymbol{q}}_{\bf{1}}$. **a** The reconstructed partial density matrix of the $| {W}_{3}^{4}\rangle$ state. Panel shows the quantum state distribution from projective measurement on *q*_1_. **b**–**d** The reconstructed partial density matrix of the remaining two ququarts after projective measurement on *q*_1_. **b** Projected onto  ∣0〉. **c** Projected onto  ∣1〉. **d** Projected onto  ∣2〉. Errors are estimated from photon Poisson statistics. Navy represents the real parts of the measured diagonal elements and non-zero off-diagonal elements of the density matrix, while beige indicates the unmeasured elements

Based on the results in Fig. 5, we partially reconstructed the real part of the density matrix for the 3-ququart W state in Fig. 6a. The fidelity of the reconstructed density matrix is *F* = 0.901 ± 0.012. As noted in Table 1, projective measurement on one ququart of the $| {W}_{3}^{4}\rangle$ state results in different states for the remaining two ququarts–with different probabilities–depending on the measurement outcome. We measured its distribution after projecting *q*_1_, and the results presented in the inset of Fig. 6a match Table 1 well at *n* = 3 and *d* = 4, with a distribution fidelity of *F*_*s*_ = 0.998 ± 0.013.

Furthermore, we conducted projective measurements on *q*_1_ and characterized the remaining states. Using the same approach, we measured the diagonal elements and real parts of non-zero off-diagonal elements in the density matrices of $| {W}_{2}^{4}\rangle$, $| {W}_{2}^{3}\rangle$, and $| {W}_{2}^{2}\rangle$, as shown in Fig. 6b–d. The corresponding quantum state fidelities are 0.934 ± 0.024, 0.927 ± 0.020, and 0.979 ± 0.027, respectively–all exceeding the lower bounds of 3/4, 2/3, and 1/2. This validates the prediction in Table 1 that qudit W states exhibit stronger robustness: at *n* = 3, *d* = 4, the probability (1/10 in theory, measured to be 0.109 ± 0.004) of entanglement loss upon losing one ququart is lower than that (1/3) in the two-dimensional case.

## Discussion

We propose a new generalization of W states in high dimensions and demonstrate that it exhibits stronger robustness than its two-dimensional counterparts. Specifically, the probability of losing entanglement upon a qudit loss in an *n*-qudit W state is $1/{C}_{d+n-2}^{n-1}$, significantly lower than the 1/*n* probability for *d* = 2. We also introduce linearly scalable qudit quantum circuits and an efficient photonic scheme for generating these states and qudit GHZ states. Using a programmable high-dimensional entanglement generator on a two-photon silicon-photonic chip, we experimentally generate and measure the 3-ququart W and GHZ states via the proposed circuits and scheme. The results show high fidelities, verifying the genuine entanglement of the GHZ state and enhanced robustness of the qudit W state.

Our proposed theoretical and experimental schemes enable the programmable realization of photonic entangled states with arbitrary dimensions and qudit numbers, including qudit GHZ, linear Cluster, and W states. This offers an efficient shortcut for generating large-scale qudit entangled states^38,41,42,47^. Experimentally, we realize the unprecedented 3-ququart GHZ and W states using two photons and one ancillary qudit, with fidelities significantly higher than prior similar-scale results^16^. This result provides a promising platform for the research and application of large-scale qudit entangled states, paving the way for potentially interesting quantum applications^48–52^.

## Methods

Additional details – including the definition and discussion of qudit Dicke states, the robustness comparison of qudit W states, the generalized quantum circuit for the (*n* + 2)-qudit W state, the scalability analysis of the on-chip entanglement generator, the device fabrication process, the detailed experimental setup, and the characterization methods for 4-dimensional entangled states – are provided in the Supplementary Information (Supplementary Appendices A–G).

## Supplementary information

Appendices A-G of the main text

## Data Availability

The data supporting this paper’s plots and other study findings are available from the corresponding author upon reasonable request.
