# Supplementary material for: Qudit W and Greenberger–Horne–Zeilinger states on a two-photon chip
Source: Light Sci Appl. 2026 Aug 5;15:340. doi: 10.1038/s41377-026-02285-7 (PMC13443087; doi:10.1038/s41377-026-02285-7)
Supplement: Supplementary file 1 [file 41377_2026_2285_MOESM1_ESM.pdf]

Supplementary Information: Qudit W and Greenberger-Horne-Zeilinger States on a Two-photon Chip

Yulin Chi[1,2,*], Haiyu Ding[1], Fei Wang[1], Chengkang Pan[1], Chunju Shao[1]

[1] *China Mobile Research Institute, China Mobile, 32 West Xuanwumen Street, Beijing, 100053, China.*
[2] *State Key Laboratory for Mesoscopic Physics, School of Physics, Peking University, 209 Chengfu Road, Beijing, 100871, China.*

*Email(s): chiyulin@chinamobile.com

Appendix A The Qudit Dicke States

Qudit Dicke states are equal-weight superposition n-qudit states with k_0 $|0\rangle$'s, k_1 $|1\rangle$'s, ..., and k_{d-1} $|d-1\rangle$'s, which satisfy $\sum_{i=0}^{d-1} k_i = n$. Their quantum states can be expressed as [43]:

$$|\mathrm{D}_n(k_0, k_1, \dots, k_{d-1})\rangle = \frac{1}{\sqrt{\binom{n}{k_0, k_1, \dots, k_{d-1}}}} \sum_{\omega \in \mathcal{P}_{M(k_0, k_1, \dots, k_{d-1})}} |\omega\rangle \tag{S.1}$$

where $\binom{n}{k_0, k_1, \dots, k_{d-1}} = \frac{n!}{\prod_{j=0}^{d-1} k_j!}$ and $\mathcal{P}_{M(k_0, k_1, \dots, k_{d-1})}$ is the set of permutations of the multiset:

$$M(k_0, k_1, \dots, k_{d-1}) = \{\underbrace{0, \dots, 0}_{k_0}, \underbrace{1, \dots, 1}_{k_1}, \dots, \underbrace{d-1, \dots, d-1}_{k_{d-1}}\} \tag{S.2}$$

In the $d = 2$ case, we have:

$$|\mathrm{D}_n(n-1, 1)\rangle = \frac{1}{\sqrt{n}} \left(|00\dots001\rangle + |00\dots010\rangle + \dots + |10\dots000\rangle\right) = |\mathrm{W}_n^2\rangle \tag{S.3}$$

That is, W states are a type of Dicke states. Similarly, in the $d > 2$ case, we can also obtain:

$$|\mathrm{D}_n(k_i = n-1, k_j = 1)\rangle = \frac{1}{\sqrt{n}} \left(|ii\dots iij\rangle + |ii\dots iji\rangle + \dots + |ji\dots iii\rangle\right) \tag{S.4}$$

This state is also equivalent to the quantum state $|\mathrm{W}_n^2\rangle$ under single-qudit rotations.

Beyond this class of Dicke states, other types of Dicke states may also be candidates for qudit W states. We first restrict our discussion to the case of $n = 3$. When $d = 3$, an evenly distributed qudit Dicke state can be written as:

$$|\mathrm{D}_3(1,1,1)\rangle = \frac{1}{\sqrt{6}}\left(|012\rangle + |021\rangle + |102\rangle + |120\rangle + |201\rangle + |210\rangle\right) \tag{S.5}$$

It can be seen that after measuring one of the qudits, the remaining two qudits remain an entangled state; thus, it also exhibits the properties of W states. However, when $d > 3$, similar qudit Dicke states are given by:

$$|\mathrm{D}_3(k_i = 1, k_j = 1, k_l = 1)\rangle = \frac{1}{\sqrt{6}}\left(|ijl\rangle + |ilj\rangle + |jil\rangle + |jli\rangle + |lij\rangle + |lji\rangle\right) \tag{S.6}$$

This implies that when d is further increased to values greater than 3, the resulting qudit Dicke states remain equivalent to those when $d = 3$ under single-qudit rotations. For the n-qudit case, when $d > n$:

$$|\mathrm{D}_n(k_{i_1} = k_{i_2} = \cdots = k_{i_n} = 1)\rangle = \frac{1}{\sqrt{n!}} \sum_{\omega \in \mathcal{P}_{\{i_1, i_2, \ldots, i_n\}}} |\omega\rangle \tag{S.7}$$

where $i_1, i_2, \ldots, i_n \in \{0, 1, \ldots, d-1\}$ and $|\{i_1, i_2, \ldots, i_n\}| = n$. This equation holds for all qudit Dicke states where n of the k_i $(i = 0, 1, \ldots, d-1)$ are equal to 1. It implies that this class of quantum states depends only on n, with their representations for $d > n$ equivalent to those for $d = n$ under single-qudit rotations.

In general, consider the qudit Dicke state $|\mathrm{D}_n(k_0, k_1, \ldots, k_{d-1})\rangle$, where $k_{i_1}, k_{i_2}, \ldots, k_{i_r} \neq 0$ and r is the number of non-zero values in $\{k_0, k_1, \ldots, k_{d-1}\}$ (thus satisfying $r \leq n$). We find that $|\mathrm{D}_n(k_0, k_1, \ldots, k_{d-1})\rangle$ at $d > r$ is equivalent to $|\mathrm{D}_n(k_{i_1}, k_{i_2}, \ldots, k_{i_r})\rangle$ at $d = r$ under single-qudit rotations. Thus, when using qudit Dicke states as the high-dimensional generalization of W states, they perform well only for $d \leq r \leq n$ — a condition not satisfied in our experiment $(d = 4, n = 3)$ — and are equivalent to those at $d = r$ when $d > r$. For example, when $d = 4, n = 3$, the

Dicke states are:

$$
\begin{aligned}
|\mathrm{D}_3(1,1,1,0)\rangle &= \frac{1}{\sqrt{6}}\left(|012\rangle + |021\rangle + |102\rangle + |120\rangle + |201\rangle + |210\rangle\right) \\
|\mathrm{D}_3(1,1,0,1)\rangle &= \frac{1}{\sqrt{6}}\left(|013\rangle + |031\rangle + |103\rangle + |130\rangle + |301\rangle + |310\rangle\right) \\
|\mathrm{D}_3(1,0,1,1)\rangle &= \frac{1}{\sqrt{6}}\left(|023\rangle + |032\rangle + |203\rangle + |230\rangle + |302\rangle + |320\rangle\right) \\
|\mathrm{D}_3(0,1,1,1)\rangle &= \frac{1}{\sqrt{6}}\left(|123\rangle + |132\rangle + |213\rangle + |231\rangle + |312\rangle + |321\rangle\right) \\
|\mathrm{D}_3(2,1,0,0)\rangle &= \frac{1}{\sqrt{3}}\left(|001\rangle + |010\rangle + |100\rangle\right) \\
|\mathrm{D}_3(2,0,1,0)\rangle &= \frac{1}{\sqrt{3}}\left(|002\rangle + |020\rangle + |200\rangle\right) \\
&\vdots
\end{aligned}
\tag{S.8}
$$

It can be observed that all of them possess at least one unused energy level.

Appendix B Robustness Comparison of the Qudit W State

According to the definition of the n-qudit W state $|\mathrm{W}_n^d\rangle$ in the main text, we can obtain:

$$
\begin{aligned}
|\mathrm{W}_n^d\rangle = |0\rangle \sqrt{\frac{C_{d+n-3}^{n-2}}{C_{d+n-2}^{n-1}}}\, |\mathrm{W}_{n-1}^d\rangle + |1\rangle \sqrt{\frac{C_{d+n-4}^{n-2}}{C_{d+n-2}^{n-1}}}\, |\mathrm{W}_{n-1}^{d-1}\rangle + \cdots \\
+ |d-2\rangle \sqrt{\frac{C_{n-1}^{n-2}}{C_{d+n-2}^{n-1}}}\, |\mathrm{W}_{n-1}^{2}\rangle + |d-1\rangle \sqrt{\frac{1}{C_{d+n-2}^{n-1}}}\, |\mathrm{W}_{n-1}^{1}\rangle
\end{aligned}
\tag{S.9}
$$

The probabilities of obtaining the measurement outcomes $|0\rangle, |1\rangle, \ldots, |d-1\rangle$ and the corresponding quantum states of the remaining qudits are summarized in Tab.S1 (Tab.1 in the main text).

Taking $|\mathrm{W}_3^d\rangle$ and $|\mathrm{W}_n^4\rangle$ as examples, we calculated the probability distributions of the quantum state after losing one qudit for different d and n, respectively. The results are shown in Fig.S1(a,b), where the red curves represent the probability of the quantum state losing entanglement. As observed, this probability decreases significantly with the increase of dimension d. Furthermore, Fig.S1(c) compares the probability of $|\mathrm{W}_n^d\rangle$ losing entanglement (resulting $|\mathrm{W}_{n-1}^1\rangle = |0\rangle^{\otimes(n-1)}$) after single-qudit loss versus n for different d. It is evident that increasing d enhances the quantum state's

Table S1 Post-measurement state statistics after single-qudit measurement on $|\mathrm{W}_n^d\rangle$

Proabbility	Measurement result	Post-measurement state
$1/C_{d+n-2}^{n-1}$	$\|d-1\rangle$	$\|\mathrm{W}_{n-1}^1\rangle$
$C_{n-1}^{n-2}/C_{d+n-2}^{n-1}$	$\|d-2\rangle$	$\|\mathrm{W}_{n-1}^2\rangle$
$\vdots$	$\vdots$	$\vdots$
$C_{d+n-4}^{n-2}/C_{d+n-2}^{n-1}$	$\|1\rangle$	$\|\mathrm{W}_{n-1}^{d-1}\rangle$
$C_{d+n-3}^{n-2}/C_{d+n-2}^{n-1}$	$\|0\rangle$	$\|\mathrm{W}_{n-1}^{d}\rangle$

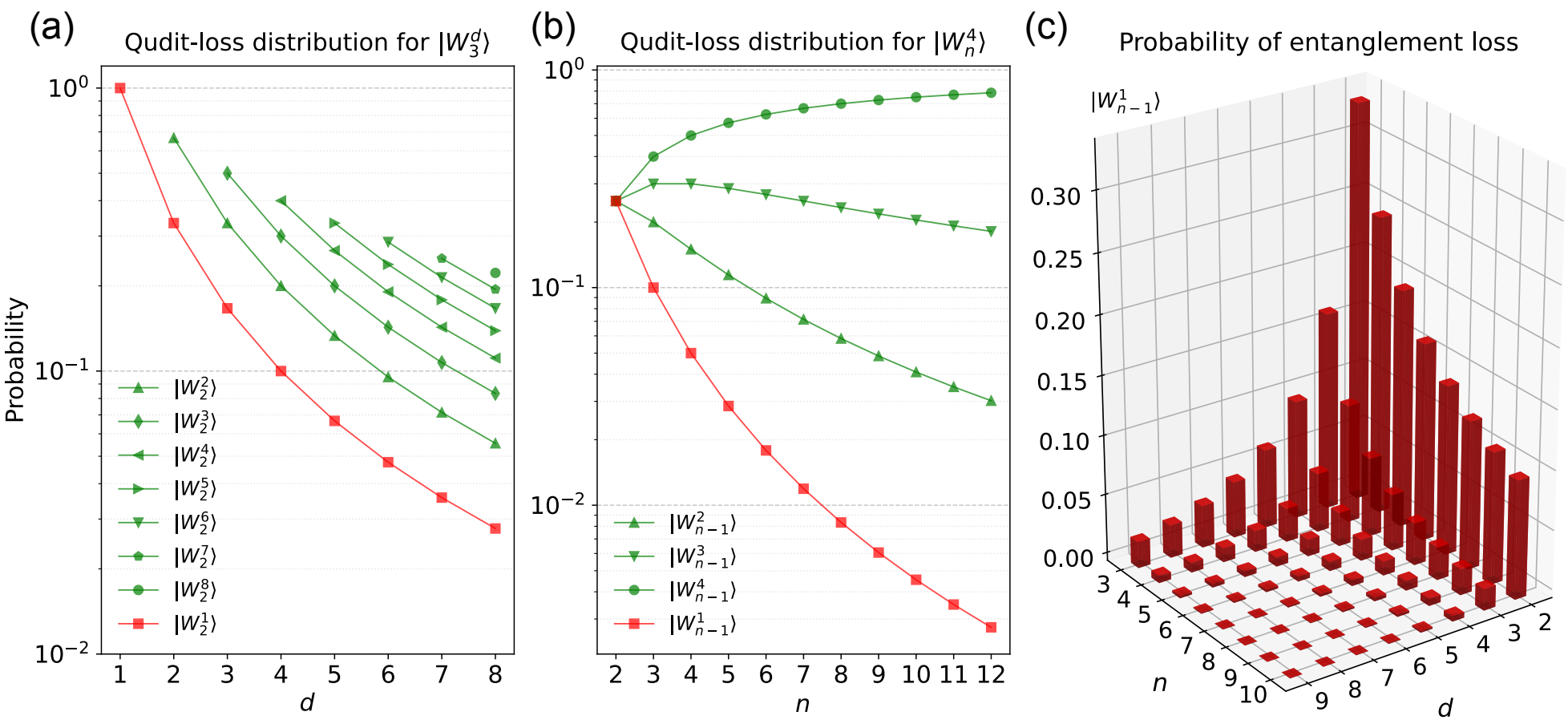

Fig. S1 (a) Probability distributions for the post-measurement states of $|\mathrm{W}_3^d\rangle$ for different d. (b) Probabilities of post-measurement states for $|\mathrm{W}_n^4\rangle$ at different n. (c) Comparison of the entanglement-losing probabilities versus n for different d in $|\mathrm{W}_n^d\rangle$. Red dots, curves and bars denote the probability of entanglement loss in the system.

robustness against loss with increasing n, satisfying:

$$\frac{1}{C_{d+n-2}^{n-1}} = \frac{(n-1)!(d-1)!}{(d+n-2)!} = \frac{1\times 2\times\cdots\times(d-1)}{n(n+1)\cdots(n+d-2)} \leq \frac{1}{n} \tag{S.10}$$

when $d=2,3,4,\ldots$ and the equality holds when $d=2$. For Eq.(S.10), when n is sufficiently large, its result can be considered asymptotically proportional to $n^{-(d-1)}$. This clearly indicates that, in addition to increasing n, increasing d can also significantly enhance the loss resistance of the W state.

Appendix C The Quantum Circuit for the $(n+2)$-qudit W State

In the main text, we provided the definition of single-qudit rotation $G_d(1/C_{d+1}^2)$ acting on $|0\rangle$ for simplicity. Here, we further generalize it to the following controlled form (acting on the initial state $|d-1\rangle|0\rangle$):

$$C_dG_d\left(\frac{1}{C_{d+1}^2}\right) = \text{Diag}\left[I_d, G_d\left(\frac{1}{C_3^2}\right), G_d\left(\frac{1}{C_4^2}\right), \cdots, G_d\left(\frac{1}{C_d^2}\right), G_d\left(\frac{1}{C_{d+1}^2}\right)\right] \tag{S.11}$$

where $G_d\left(\frac{1}{C_3^2}\right), G_d\left(\frac{1}{C_4^2}\right), \cdots, G_d\left(\frac{1}{C_d^2}\right), G_d\left(\frac{1}{C_{d+1}^2}\right)$ are defined as follows:

$$\begin{aligned}
G_d\left(\frac{1}{C_3^2}\right)|0\rangle &= \sqrt{\frac{1}{C_3^2}}|0\rangle + \sqrt{\frac{C_2^1}{C_3^2}}|1\rangle \\
G_d\left(\frac{1}{C_4^2}\right)|0\rangle &= \sqrt{\frac{1}{C_4^2}}|0\rangle + \sqrt{\frac{C_2^1}{C_4^2}}|1\rangle + \sqrt{\frac{C_3^1}{C_4^2}}|2\rangle \\
&\vdots \\
G_d\left(\frac{1}{C_d^2}\right)|0\rangle &= \sqrt{\frac{1}{C_d^2}}|0\rangle + \sqrt{\frac{C_2^1}{C_d^2}}|1\rangle + \cdots + \sqrt{\frac{C_{d-1}^1}{C_d^2}}|d-2\rangle \\
G_d\left(\frac{1}{C_{d+1}^2}\right)|0\rangle &= \sqrt{\frac{1}{C_{d+1}^2}}|0\rangle + \sqrt{\frac{C_2^1}{C_{d+1}^2}}|1\rangle + \cdots + \sqrt{\frac{C_{d-1}^1}{C_{d+1}^2}}|d-2\rangle + \sqrt{\frac{C_d^1}{C_{d+1}^2}}|d-1\rangle
\end{aligned} \tag{S.12}$$

The above generalization applies to any 3-qudit W state generation circuit described in Fig.1(b). For the generation of arbitrary $(n+2)$-qudit W states ($n = 1, 2, 3, \cdots$), we further generalize the operator expression as follows ($k = 0, 1, \cdots, n$):

$$C_dG_d\left(\frac{1}{C_{d+k}^{1+k}}\right) = \text{Diag}\left[I_d, G_d\left(\frac{1}{C_{2+k}^{1+k}}\right), G_d\left(\frac{1}{C_{3+k}^{1+k}}\right), \cdots, G_d\left(\frac{1}{C_{d+k}^{1+k}}\right)\right] \tag{S.13}$$

where we define:

$$\begin{aligned}
G_d\left(\frac{1}{C_{2+k}^{1+k}}\right)|0\rangle &= \sqrt{\frac{C_k^k}{C_{2+k}^{1+k}}}|0\rangle + \sqrt{\frac{C_{1+k}^k}{C_{2+k}^{1+k}}}|1\rangle \\
G_d\left(\frac{1}{C_{3+k}^{1+k}}\right)|0\rangle &= \sqrt{\frac{C_k^k}{C_{3+k}^{1+k}}}|0\rangle + \sqrt{\frac{C_{1+k}^k}{C_{3+k}^{1+k}}}|1\rangle + \sqrt{\frac{C_{2+k}^k}{C_{3+k}^{1+k}}}|2\rangle \\
&\vdots \\
G_d\left(\frac{1}{C_{d-1+k}^{1+k}}\right)|0\rangle &= \sqrt{\frac{C_k^k}{C_{d-1+k}^{1+k}}}|0\rangle + \sqrt{\frac{C_{1+k}^k}{C_{d-1+k}^{1+k}}}|1\rangle + \cdots + \sqrt{\frac{C_{d-2+k}^k}{C_{d-1+k}^{1+k}}}|d-2\rangle \\
G_d\left(\frac{1}{C_{d+k}^{1+k}}\right)|0\rangle &= \sqrt{\frac{C_k^k}{C_{d+k}^{1+k}}}|0\rangle + \sqrt{\frac{C_{1+k}^k}{C_{d+k}^{1+k}}}|1\rangle + \cdots + \sqrt{\frac{C_{d-2+k}^k}{C_{d+k}^{1+k}}}|d-2\rangle \\
&\quad + \sqrt{\frac{C_{d-1+k}^k}{C_{d+k}^{1+k}}}|d-1\rangle
\end{aligned} \tag{S.14}$$

We propose the linearly scalable quantum circuit shown in Fig.S2 to realize the

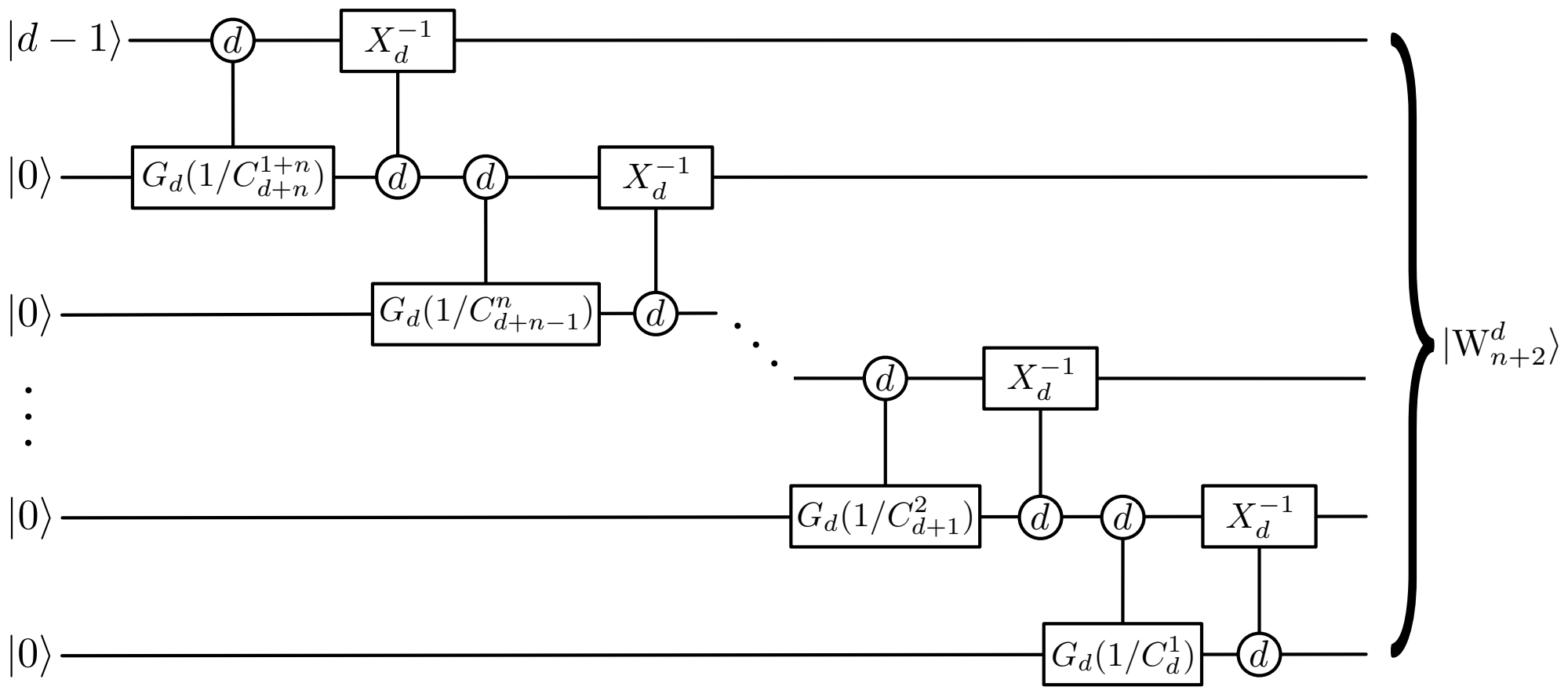

Fig. S2 The linearly scalable quantum circuit for the generation of $(n+2)$-qudit W state $|\mathrm{W}_{n+2}^d\rangle$, $n = 1, 2, 3, \ldots$

$(n+2)$-qudit W state $|\mathrm{W}_{n+2}^d\rangle$ in arbitrary d-dimensional cases, where

$$|\mathrm{W}_{n+2}^d\rangle = \frac{1}{\sqrt{C_{d+n}^{n+1}}} \sum_{i_1+i_2+\cdots+i_{n+2}=d-1} |i_1, i_2, \cdots, i_{n+2}\rangle \tag{S.15}$$

We label the $n+2$ qudits in Fig.S2 sequentially top-to-bottom as qudit $1, 2, \cdots, n+2$. The initial quantum state of the system can be written as:

$$|d-1\rangle\,|0\rangle^{\otimes(n+1)} \tag{S.16}$$

Next, we use mathematical induction to prove that the quantum circuit in Fig.S2 can generate the $|\mathrm{W}^d_{n+2}\rangle$ state.

First, when $n = 1$, this is a 3-qudit quantum circuit with the initial state $|d-1\rangle\,|0\rangle\,|0\rangle$. After the $C_dG_d(1/C^2_{d+1})$ gate, the quantum state of qudit 1 & 2 can be written as:

$$|d-1\rangle \left(\sqrt{\frac{C^1_1}{C^2_{d+1}}}\,|0\rangle + \sqrt{\frac{C^1_2}{C^2_{d+1}}}\,|1\rangle + \cdots + \sqrt{\frac{C^1_{d-1}}{C^2_{d+1}}}\,|d-2\rangle + \sqrt{\frac{C^1_d}{C^2_{d+1}}}\,|d-1\rangle\right) \tag{S.17}$$

After applying the $C_dX_d^{-1}$ gate with qudit 2 as the control and qudit 1 as the target, the quantum state evolves into:

$$\sqrt{\frac{C^1_1}{C^2_{d+1}}}\,|d-1,0\rangle + \sqrt{\frac{C^1_2}{C^2_{d+1}}}\,|d-2,1\rangle + \cdots + \sqrt{\frac{C^1_{d-1}}{C^2_{d+1}}}\,|1,d-2\rangle + \sqrt{\frac{C^1_d}{C^2_{d+1}}}\,|0,d-1\rangle \tag{S.18}$$

Following the application of the $C_dG_d(1/C^1_d)$ gate with qudit 2 as the control and qudit 3 (initialized in $|0\rangle$) as the target, the quantum state of the system can be expressed as:

$$\begin{aligned}
&\sqrt{\frac{C^1_1}{C^2_{d+1}}}\,|d-1,0\rangle\,I_d\,|0\rangle + \sqrt{\frac{C^1_2}{C^2_{d+1}}}\,|d-2,1\rangle\,G_d(1/C^1_2)\,|0\rangle + \cdots \\
&+\sqrt{\frac{C^1_{d-1}}{C^2_{d+1}}}\,|1,d-2\rangle\,G_d(1/C^1_{d-1})\,|0\rangle + \sqrt{\frac{C^1_d}{C^2_{d+1}}}\,|0,d-1\rangle\,G_d(1/C^1_d)\,|0\rangle \\
&=\sqrt{\frac{C^1_1}{C^2_{d+1}}}\,|d-1,0\rangle\,|0\rangle \\
&+\sqrt{\frac{C^1_2}{C^{1+1}_{d+1}}}\,|d-2,1\rangle\left(\sqrt{\frac{1}{C^1_2}}\,|0\rangle + \sqrt{\frac{C^0_1}{C^1_2}}\,|1\rangle\right) \\
&+\cdots \\
&+\sqrt{\frac{C^1_{d-1}}{C^2_{d+1}}}\,|1,d-2\rangle\left(\sqrt{\frac{1}{C^1_{d-1}}}\,|0\rangle + \sqrt{\frac{C^0_1}{C^1_{d-1}}}\,|1\rangle + \cdots + \sqrt{\frac{C^0_{d-2}}{C^1_{d-1}}}\,|d-2\rangle\right) \\
&+\sqrt{\frac{C^1_d}{C^2_{d+1}}}\,|0,d-1\rangle\left(\sqrt{\frac{1}{C^1_d}}\,|0\rangle + \sqrt{\frac{C^0_1}{C^1_d}}\,|1\rangle + \cdots + \sqrt{\frac{C^0_{d-2}}{C^1_d}}\,|d-2\rangle + \sqrt{\frac{C^0_{d-1}}{C^1_d}}\,|d-1\rangle\right)
\end{aligned} \tag{S.19}$$

Subsequently, after applying the $C_dX_d^{-1}$ gate with qudit 3 as the control and qudit 2 as the target, the quantum state of the system becomes:

$$
\begin{aligned}
&\sqrt{\frac{1}{C_{d+1}^2}}\,|d-1,0,0\rangle \\
&+\sqrt{\frac{1}{C_{d+1}^2}}\Big(\,|d-2,1,0\rangle+|d-2,0,1\rangle\,\Big) \\
&+\cdots \\
&+\sqrt{\frac{1}{C_{d+1}^2}}\Big(\,|1,d-2,0\rangle+|1,d-3,1\rangle+\cdots+|1,0,d-2\rangle\,\Big) \\
&+\sqrt{\frac{1}{C_{d+1}^2}}\Big(\,|0,d-1,0\rangle+|0,d-2,1\rangle+\cdots+|0,1,d-2\rangle+|0,0,d-1\rangle\,\Big) \\
&=|\mathrm{W}_3^d\rangle
\end{aligned}
\tag{S.20}
$$

Thus, we have realized the $|\mathrm{W}_3^d\rangle$ state. Next, we assume that when $n=l-1$ $(l=2,3,4,\cdots)$, the quantum circuit in Fig.S2 can generate the $|\mathrm{W}_{l+1}^d\rangle$ state $(d=2,3,4,\cdots)$. Then, when $n=l$, the initial quantum states of qudit 1 and qudit 2 are:

$$|d-1\rangle\,|0\rangle \tag{S.21}$$

After the $C_d(1/C_{d+l}^{1+l})$ gate, the quantum state of qudit 1 & 2 can be written as:

$$|d-1\rangle\left(\sqrt{\frac{C_l^l}{C_{d+l}^{1+l}}}\,|0\rangle+\sqrt{\frac{C_{1+l}^l}{C_{d+l}^{1+l}}}\,|1\rangle+\cdots+\sqrt{\frac{C_{d-2+l}^l}{C_{d+l}^{1+l}}}\,|d-2\rangle+\sqrt{\frac{C_{d-1+l}^l}{C_{d+l}^{1+l}}}\,|d-1\rangle\right) \tag{S.22}$$

After applying the $C_dX_d^{-1}$ gate with qudit 2 as the control and qudit 1 as the target, the quantum state evolves to:

$$\sqrt{\frac{C_l^l}{C_{d+l}^{1+l}}}\,|d-1,0\rangle+\sqrt{\frac{C_{1+l}^l}{C_{d+l}^{1+l}}}\,|d-2,1\rangle+\cdots+\sqrt{\frac{C_{d-2+l}^l}{C_{d+l}^{1+l}}}\,|1,d-2\rangle+\sqrt{\frac{C_{d-1+l}^l}{C_{d+l}^{1+l}}}\,|0,d-1\rangle \tag{S.23}$$

When the quantum state of qudit 2 is $|0\rangle$, it is straightforward that the subsequent quantum circuit generates the state $|0\rangle^{\otimes(l+1)}$ for qudits $2,3,4,\cdots,l+2$. When the quantum state of qudit 2 is $|1\rangle$, the subsequent circuit generates the state $|\mathrm{W}_{l+1}^2\rangle$ for qudits $2,3,4,\cdots,l+2$ based on the hypothesis. By analogy, when the quantum state of qudit 2 is $|2\rangle$, the subsequent circuit generates $|\mathrm{W}_{l+1}^3\rangle$; this pattern continues such that when qudit 2 is in the state $|d-1\rangle$, the subsequent circuit generates $|\mathrm{W}_{l+1}^d\rangle$ for qudits $2,3,4,\cdots,l+2$. Therefore, the quantum state generated by the circuit when

$n = l$ is:

$$
\begin{aligned}
&\sqrt{\frac{1}{C_{d+l}^{1+l}}}\,|d-1\rangle\,|0\rangle^{\otimes(l+1)} + \sqrt{\frac{C_{1+l}^{l}}{C_{d+l}^{1+l}}}\,|d-2\rangle\,|\mathrm{W}_{l+1}^{2}\rangle + \cdots \\
&+ \sqrt{\frac{C_{d-2+l}^{l}}{C_{d+l}^{1+l}}}\,|1\rangle\,|\mathrm{W}_{l+1}^{d-1}\rangle + \sqrt{\frac{C_{d-1+l}^{l}}{C_{d+l}^{1+l}}}\,|0\rangle\,|\mathrm{W}_{l+1}^{d}\rangle \\
=&\sqrt{\frac{1}{C_{d+l}^{1+l}}}\,|d-1\rangle\,|0\rangle^{\otimes(l+1)} \\
&+ \sqrt{\frac{1}{C_{d+l}^{1+l}}}\,|d-2\rangle \sum_{i_1+i_2+\cdots+i_{l+1}=1} |i_1, i_2, \cdots, i_{l+1}\rangle + \cdots \\
&+ \sqrt{\frac{1}{C_{d+l}^{1+l}}}\,|1\rangle \sum_{i_1+i_2+\cdots+i_{l+1}=d-2} |i_1, i_2, \cdots, i_{l+1}\rangle \\
&+ \sqrt{\frac{1}{C_{d+l}^{1+l}}}\,|0\rangle \sum_{i_1+i_2+\cdots+i_{l+1}=d-1} |i_1, i_2, \cdots, i_{l+1}\rangle = |\mathrm{W}_{l+2}^{d}\rangle
\end{aligned}
\tag{S.24}
$$

Thus, if the quantum circuit depicted in Fig.S2 can generate the $|\mathrm{W}_{l+1}^{d}\rangle$ state when $n = l - 1$, it follows that the circuit produces the $|\mathrm{W}_{l+2}^{d}\rangle$ state when $n = l$. By mathematical induction, we conclude that the quantum circuit in Fig.S2 is capable of generating the $|\mathrm{W}_{n+2}^{d}\rangle$ state for any positive integer n.

Appendix D Scalability of the Entanglement Generator

Fig.S3(b) enables scaling of the entanglement generator, with the bracketed structure cascaded n times to realize Fig.S3(a). The linear quantum circuit in Fig.S3(a) can be programmed to efficiently generate the $(n+2)$-ququart GHZ, Cluster, and W states. In Fig.S3(b), by tunning the AMZIs, the 4 integrated photon-pair sources can generate a 4-dimensional Bell-type state (as q_1 & q_2):

$$
a_0\,|30\rangle + a_1\,|21\rangle + a_2\,|12\rangle + a_3\,|03\rangle \tag{S.25}
$$

According to the analysis in the main text, the structure within the square brackets can encode an ancillary ququart $i+2$ (initialized in $|0\rangle$) onto ququart $i+1$ ($i = 1, 2, \cdots, n$), perform a $C_4U(i+1)$ operation with ququart $i+1$ as the control and ququart $i+2$ as the target, then execute a C_4V operation with ququart $i+2$ as the control and ququart $i+1$ as the target, followed by a projective measurement M_{i+1} in an arbitrary basis on ququart $i+1$, retaining the quantum state of ququart $i+2$ after the measurement. R can be implemented using the dashed box ❸. Thus, we can realize the linear quantum circuit in Fig.S3(a).

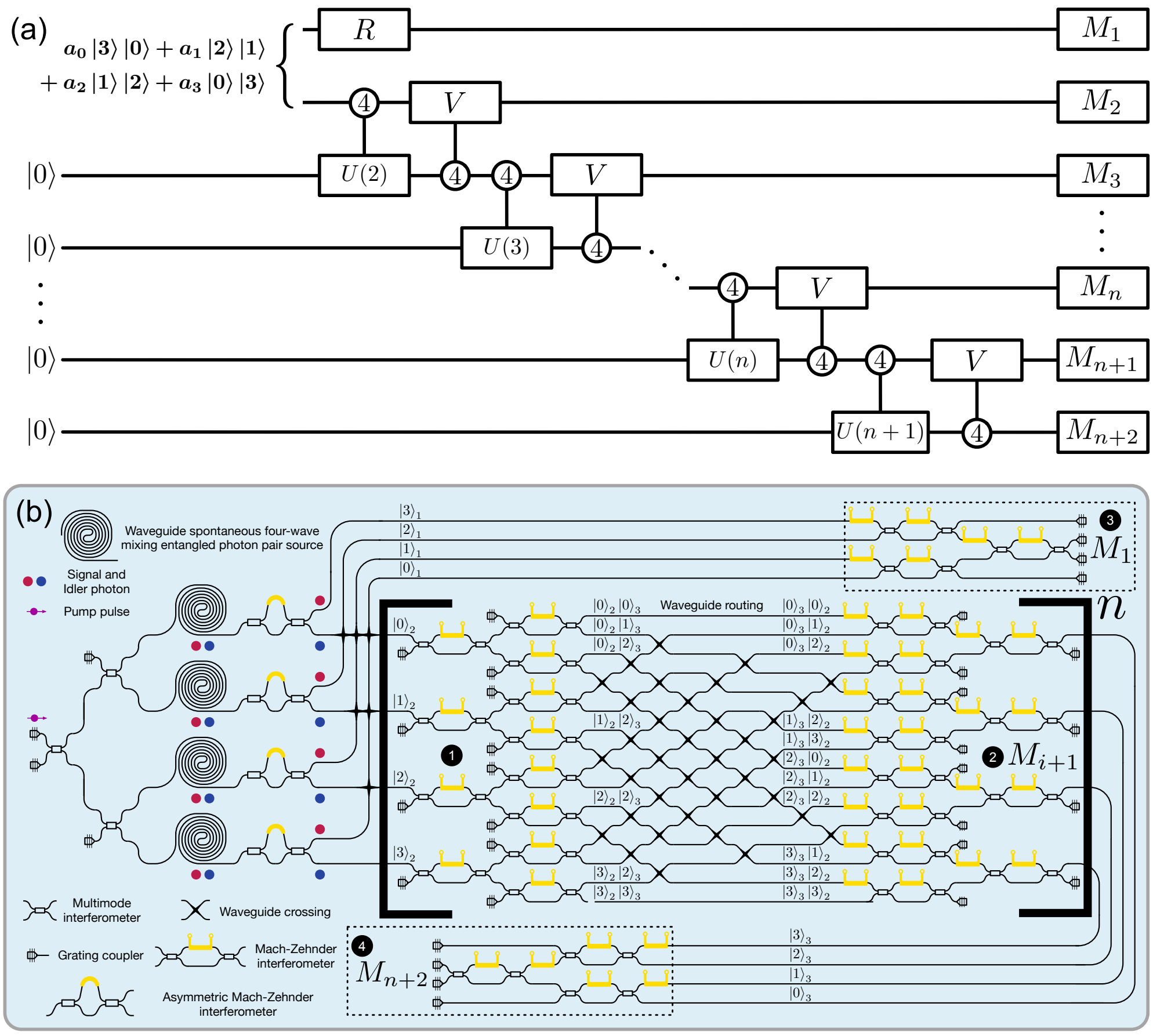

Fig. S3 (a) The linear quantum circuit for the generation of $(n+2)$-ququart entangled states including GHZ, Cluster, and W states. (b) The integrated photonic structure that can be programmed to implement the quantum circuit in (a). The bracketed structure is repeated n times with $i = 1, 2, \ldots, n$.

For the ququart GHZ state, in Fig.S3(a), we set $a_0 = a_1 = a_2 = a_3 = \frac{1}{2}$, $C_4U(2) = C_4U(3) = \cdots = C_4U(n+1) = C_4X_4$, $C_4V = \mathrm{Diag}[I_4, I_4, I_4, I_4]$, and $R = \mathrm{AntiDiag}[1,1,1,1]$. The $(n+2)$-ququart GHZ state can be generated. For the ququart Cluster state, we reprogram $R = F_4\mathrm{AntiDiag}[1,1,1,1]$ where $F_4\left|k\right\rangle = \sum_{j=0}^{3}(-1)^{jk/2}\left|j\right\rangle/2$ to change the initial entangled state into:

$$\left|\mathrm{Cluster}_2^4\right\rangle = \frac{1}{2}\left(\left|+\right\rangle\left|0\right\rangle + \left|+\mathrm{i}\right\rangle\left|1\right\rangle + \left|-\right\rangle\left|2\right\rangle + \left|-\mathrm{i}\right\rangle\left|3\right\rangle\right) \tag{S.26}$$

where $\left|+\right\rangle = F_4\left|0\right\rangle$, $\left|+\mathrm{i}\right\rangle = F_4\left|1\right\rangle$, $\left|-\right\rangle = F_4\left|2\right\rangle$ and $\left|-\mathrm{i}\right\rangle = F_4\left|3\right\rangle$. Then, by replacing the initial state $\left|0\right\rangle$ of ququart $3, 4, \ldots, n+2$ by $\left|+\right\rangle$, and reprogramming

$C_4U(2) = C_4U(3) = \cdots = C_4U(n+1) = C_4Z_4 = \text{Diag}[I_4, Z_4, Z_4^2, Z_4^3]$ where $Z_4 = \text{Diag}[1, \text{i}, -1, -\text{i}]$, the $(n+2)$-ququart linear Cluster state can also be generated [51].

For the ququart W state, it can be realized using the architecture in Fig.S3(a,b) by reconfiguring a_0, a_1, a_2, a_3, and $R = I_4$ to prepare the initial state as:

$$\sqrt{\frac{C_n^n}{C_{n+4}^{n+1}}}|30\rangle + \sqrt{\frac{C_{n+1}^n}{C_{n+4}^{n+1}}}|21\rangle + \sqrt{\frac{C_{n+2}^n}{C_{n+4}^{n+1}}}|12\rangle + \sqrt{\frac{C_{n+3}^n}{C_{n+4}^{n+1}}}|03\rangle \tag{S.27}$$

Following Fig.S2 with $d = 4$, the i-th layer operations in Fig.S3(b) are programmed as: $C_4U(i+1) = C_4G_4(1/C_{4+n-i}^{n+1-i})$ and $C_4V = C_4X_4^{-1}$, thereby enabling the generation and projective measurement of $|\text{W}_{n+2}^4\rangle$.

Appendix E Device Fabrication

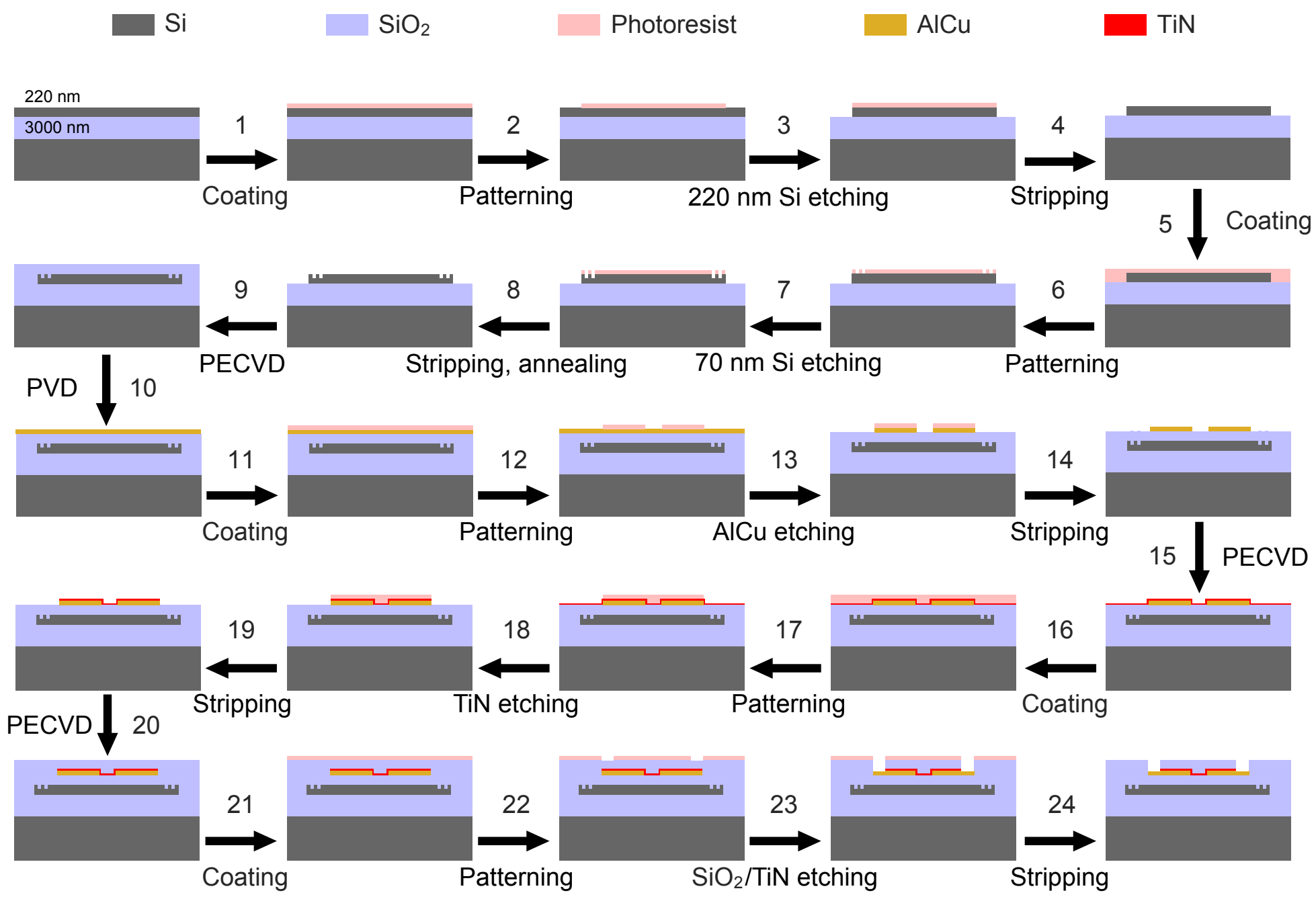

Fig. S4 Schematic of the 24-step CMOS fabrication flow for the silicon-photonic 3-ququart entanglement generator in Fig.2(e).

Our silicon-photonic chip consists of 71 multimode interferometers, 52 phase modulators, 4 photon-pair sources, 42 waveguide crossings and 36 grating couplers. It was fabricated using the standardized complementary metal oxide semiconductor (CMOS) process, as shown in Fig.S4, with 24 steps and detailed below in 5 parts:

1. Substrate Preparation (step 1): An 8-inch silicon-on-insulator (SOI) wafer with a 220-nm silicon device layer and 3-μm buried oxide was employed as the starting material. A photoresist layer was spin-coated onto the wafer surface for lithography and pattern transfer.

2. Waveguide Fabrication (steps 2-8): Deep ultraviolet (DUV) lithography at 248 nm was employed to pattern the waveguide structures on the photoresist. Single-mode waveguides with a nominal width of 450 nm were defined, with strategic widening to 4 μm in long and straight segments to minimize transmission losses. The pattern was transferred to the silicon layer via dual-step inductively coupled plasma (ICP) etching, forming both deep-etched (220 nm) and shallow-etched (70 nm) structures, sequentially through two separate coating-patterning-etching-stripping steps:

- Deep-etched waveguides were utilized for SFWM photon sources (1.2 cm length), multimode interferometer beam splitters, and thermo-optic phase shifters.
- Shallow-etched waveguides were reserved for crossing structures and grating couplers, followed by annealing to smooth the waveguide surfaces.

3. Dielectric and Metal Deposition (steps 9-10): A 1-μm SiO_2 cladding layer was deposited via plasma-enhanced chemical vapor deposition (PECVD) to isolate the waveguides. A multilayer metal stack (AlCu layer) was then deposited using physical vapor deposition (PVD):

- Ti (10 nm) as an adhesion layer
- TiN (20 nm) as a diffusion barrier
- AlCu (800 nm) as the primary conductor
- TiN (20 nm) as an anti-reflective coating

4. Electrode Fabrication (steps 11-19): The metal layer was patterned using DUV lithography (coating + patterning), selective metal etching, and stripping to form electrical interconnects and electrodes for thermo-optic tuning. A secondary 50-nm TiN layer, deposited via PECVD, was also patterned using DUV lithography, selective TiN etching, and stripping to create localized heating elements for phase modulation.

5. Final Passivation (steps 20-24): A 1-μm SiO_2 top layer was deposited via PECVD for protection. Bonding pads were opened via DUV lithography, selective etching of the SiO_2/TiN stack, and stripping to expose AlCu contact areas for wire bonding.

This fabrication approach ensures compatibility with commercial CMOS processes while enabling precise control over optical and electrical properties, critical for scalable quantum photonic applications.

Appendix F Experimental Setup

Fig. S5 shows a schematic of the experimental testing platform, which consists of two parts: optical and electrical.

We first describe the optical part. The pump source is a 4 mW 1550.12 nm continuous-wave laser (CW Laser, model EXFO T100S-HP), which is amplified to ~150 mW via an erbium-doped fiber amplifier (EDFA, model PriTel SPFA-23-IO). To

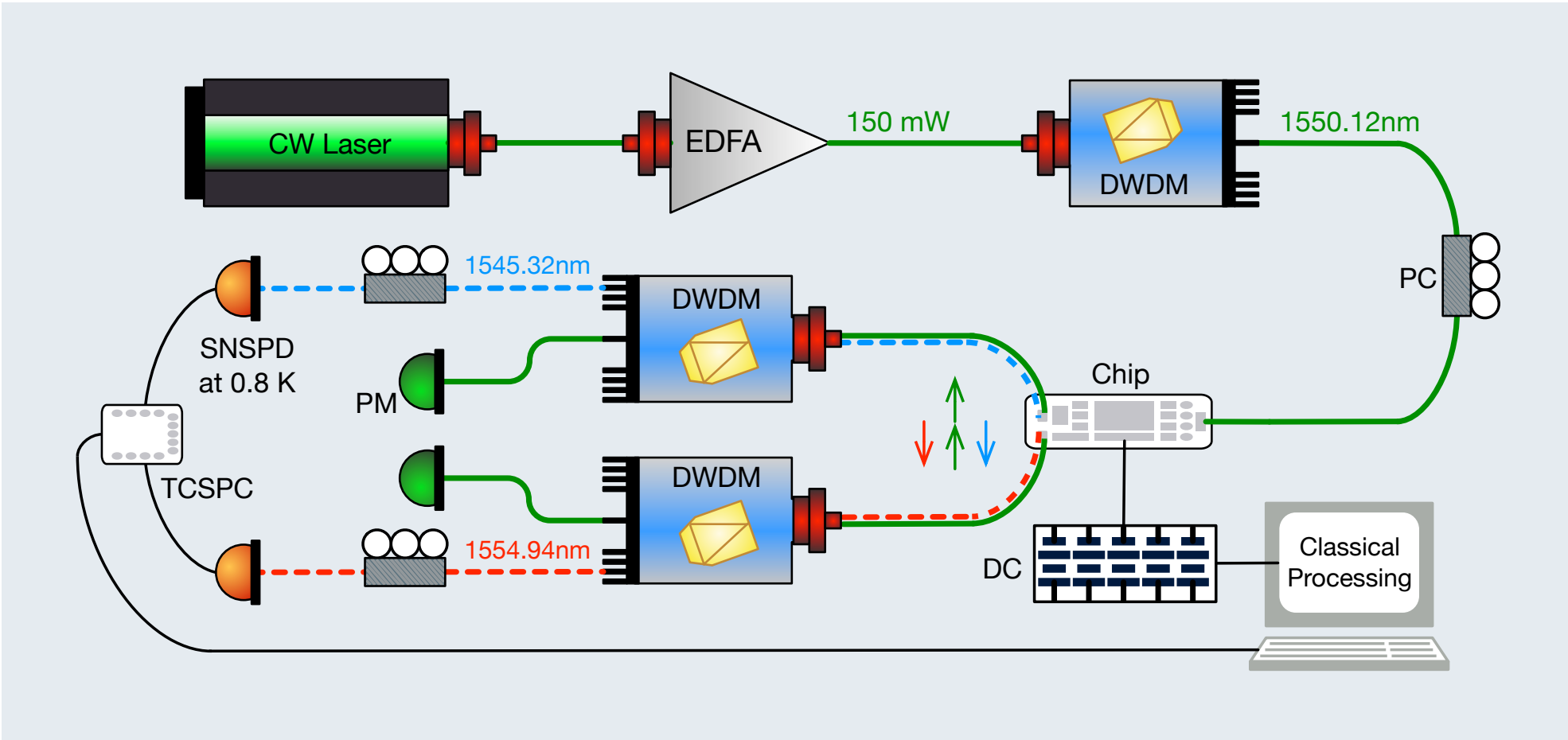

Fig. S5 Schematic of the integrated photonic chip testing system. Green solid lines: 1550.12 nm pump light; red/blue dashed lines: 1554.94 nm/1545.32 nm single photons; black solid lines: electrical signal connections. CW Laser: Continuous-wave Laser; EDFA: Erbium-Doped Fiber Amplifier; DWDM: Dense Wavelength Division Multiplexer; PC: Fiber Polarization Controller; PM: Fiber Power Meter; SNSPD: Superconducting Nanowire Single-Photon Detector; TCSPC: Time-Correlated Single Photon Counting; DC: Direct Current Voltage Source. Four arrows left of the chip illustrate the on-chip spontaneous four-wave mixing process generating photon-pairs.

filter out the co-amplified noise signals, it is passed through a dense wavelength division multiplexing (DWDM, model OPENTI DWDM-D-2-16-18-900-1-1-FA) module with an isolation greater than 100 dB, selecting the channel with a central wavelength of 1550.12 nm and a bandwidth of ∼1.2 nm. After being adjusted to the optimal polarization via a fiber polarization controller (PC, model FIBERPRO PC1100-15-F/A), the pump is coupled into the chip from a single-mode fiber via an on-chip grating coupler (with a coupling loss of ∼4 dB port^{-1}). After on-chip quantum state evolution (with on-chip waveguide transmission loss ∼3 dB cm^{-1} and multimode interferometer loss ∼0.2 dB element^{-1}), the output signal and idler photons are coupled out of the chip via on-chip grating couplers into two single-mode fibers. They contain both single photons and residual pump light; thus, they are respectively passed through a DWDM module to separate the channels with wavelengths of 1545.32 nm/1554.94 nm (bandwidth ∼1.2 nm) as single-photon channels. The residual pump light is output to a fiber power meter (PM, model THORLABS S154C) through the 1550.12 nm channel to monitor power fluctuations. The two single-photon channels are respectively adjusted to the optimal polarization via a PC and then input to superconducting nanowire single-photon detectors (SNSPDs, model Photon Spot Cryospot 4, with a detection efficiency of ∼85%), where the single-photon signals are converted into electrical pulse signals.

Next, we describe the electrical part. A large number of single-photon trigger events are observed in the electrical output of the SNSPDs. To perform real-time monitoring of two-photon coincidence events in these data streams and extract quantum information, a Time-Correlated Single Photon Counting (TCSPC, model Swabian Instruments

Time Tagger Ultra) device is employed. For the two electrical pulse channels from the SNSPDs, signals exceeding 200 mV are amplified to 5 V and then input to the TCSPC. The TCSPC calculates the photon counts and communicates with the computer via USB. The two-photon coincidence rate measured experimentally reaches the kilohertz order. The chip is mounted on a custom-made printed circuit board (PCB), placed on a water-cooled thermostatic stage, and connected to the PCB electrodes via gold wires (as shown in Fig.2(d)). The PCB is connected to DC voltage sources through fine-pitch flat flexible cables (FFCs). A commercial Qontrol Q8iv driver system is used as the DC source, and it communicates with the computer via USB to adjust the voltage applied to the on-chip thermo-optic phase modulators.

Appendix G Characterization Methods for 4-dimensional Entangled States

We first define the 6 symmetric Gell-Mann matrices (GMMs) in the four-dimensional case [52] as follows:

$$\begin{aligned} G_s^{01} &= \begin{bmatrix} 0&1&0&0\\1&0&0&0\\0&0&0&0\\0&0&0&0 \end{bmatrix}, G_s^{02} = \begin{bmatrix} 0&0&1&0\\0&0&0&0\\1&0&0&0\\0&0&0&0 \end{bmatrix}, G_s^{03} = \begin{bmatrix} 0&0&0&1\\0&0&0&0\\0&0&0&0\\1&0&0&0 \end{bmatrix} \\ G_s^{12} &= \begin{bmatrix} 0&0&0&0\\0&0&1&0\\0&1&0&0\\0&0&0&0 \end{bmatrix}, G_s^{13} = \begin{bmatrix} 0&0&0&0\\0&0&0&1\\0&0&0&0\\0&1&0&0 \end{bmatrix}, G_s^{23} = \begin{bmatrix} 0&0&0&0\\0&0&0&0\\0&0&0&1\\0&0&1&0 \end{bmatrix} \end{aligned} \tag{S.28}$$

And the 6 antisymmetric GMMs as follows:

$$\begin{aligned} G_a^{01} &= \begin{bmatrix} 0&-\mathrm{i}&0&0\\\mathrm{i}&0&0&0\\0&0&0&0\\0&0&0&0 \end{bmatrix}, G_a^{02} = \begin{bmatrix} 0&0&-\mathrm{i}&0\\0&0&0&0\\\mathrm{i}&0&0&0\\0&0&0&0 \end{bmatrix}, G_a^{03} = \begin{bmatrix} 0&0&0&-\mathrm{i}\\0&0&0&0\\0&0&0&0\\\mathrm{i}&0&0&0 \end{bmatrix} \\ G_a^{12} &= \begin{bmatrix} 0&0&0&0\\0&0&-\mathrm{i}&0\\0&\mathrm{i}&0&0\\0&0&0&0 \end{bmatrix}, G_a^{13} = \begin{bmatrix} 0&0&0&0\\0&0&0&-\mathrm{i}\\0&0&0&0\\0&\mathrm{i}&0&0 \end{bmatrix}, G_a^{23} = \begin{bmatrix} 0&0&0&0\\0&0&0&0\\0&0&0&-\mathrm{i}\\0&0&\mathrm{i}&0 \end{bmatrix} \end{aligned} \tag{S.29}$$

The last 3 diagonal GMMs are as follows:

$$G_4^1 = \begin{bmatrix} 1&0&0&0\\0&-1&0&0\\0&0&0&0\\0&0&0&0 \end{bmatrix}, G_4^2 = \frac{1}{\sqrt{3}} \begin{bmatrix} 1&0&0&0\\0&1&0&0\\0&0&-2&0\\0&0&0&0 \end{bmatrix}, G_4^3 = \frac{1}{\sqrt{6}} \begin{bmatrix} 1&0&0&0\\0&1&0&0\\0&0&1&0\\0&0&0&-3 \end{bmatrix} \tag{S.30}$$

The definition of density matrix fidelity in the main text enables the fidelity of GHZ or W states to be determined by measuring only the non-zero elements in the real part of the target state's density matrix [16]. Taking the Bell entangled state as an example, its ideal and measured density matrices are:

$$\rho_0 = \frac{1}{2}\begin{bmatrix} 1 & 0 & 0 & 1 \\ 0 & 0 & 0 & 0 \\ 0 & 0 & 0 & 0 \\ 1 & 0 & 0 & 1 \end{bmatrix}, \quad \rho = \begin{bmatrix} \rho_{00} & \rho_{01} & \rho_{02} & \rho_{03} \\ \rho_{01}^* & \rho_{11} & \rho_{12} & \rho_{13} \\ \rho_{02}^* & \rho_{12}^* & \rho_{22} & \rho_{23} \\ \rho_{03}^* & \rho_{13}^* & \rho_{23}^* & \rho_{33} \end{bmatrix} \tag{S.31}$$

We have $\mathrm{Tr}[\rho\rho_0] = (\rho_{00} + \rho_{03}^* + \rho_{03} + \rho_{33})/2 = [\rho_{00} + 2\mathrm{Re}(\rho_{03}) + \rho_{33}]/2$. Thus, we only need to measure the real part of ρ_{00}, ρ_{33} and ρ_{03} to obtain the fidelity.

G.1 Off-diagonal element measurement of 3-ququart GHZ state density matrix

The three-ququart GHZ state can be written as:

$$|\mathrm{GHZ}_3^4\rangle = \frac{1}{2}\left(|000\rangle + |111\rangle + |222\rangle + |333\rangle\right) \tag{S.32}$$

The corresponding density matrix ρ contains 4 non-zero diagonal elements $\langle iii|\,\rho\,|iii\rangle$ and 12 non-zero off-diagonal elements $\langle jjj|\,\rho\,|iii\rangle$, where $i, j = 0, 1, 2, 3$ and $i \neq j$. Once the real parts of these non-zero elements are measured, we can derive the fidelity of the density matrix corresponding to the target quantum state.

By performing projective measurements in all computational bases, we can obtain all 64 diagonal elements of the density matrix, including terms of the form $\langle iii|\,\rho\,|iii\rangle$. Thus, the key to the problem lies in measuring the 6 upper off-diagonal elements of the density matrix. We define the coherence observables:

$$\begin{aligned} \Omega_\theta^{\otimes 3}(0,1) &= (\cos\theta G_s^{01} + \sin\theta G_a^{01})^{\otimes 3}, \quad \Omega_\theta^{\otimes 3}(0,2) = (\cos\theta G_s^{02} + \sin\theta G_a^{02})^{\otimes 3} \\ \Omega_\theta^{\otimes 3}(0,3) &= (\cos\theta G_s^{03} + \sin\theta G_a^{03})^{\otimes 3}, \quad \Omega_\theta^{\otimes 3}(1,2) = (\cos\theta G_s^{12} + \sin\theta G_a^{12})^{\otimes 3} \\ \Omega_\theta^{\otimes 3}(1,3) &= (\cos\theta G_s^{13} + \sin\theta G_a^{13})^{\otimes 3}, \quad \Omega_\theta^{\otimes 3}(2,3) = (\cos\theta G_s^{23} + \sin\theta G_a^{23})^{\otimes 3} \end{aligned} \tag{S.33}$$

The eignstates of the observable $\Omega_\theta(i,j) = \cos\theta G_s^{ij} + \sin\theta G_a^{ij}$ is

$$\frac{|i\rangle \pm e^{\mathrm{i}\theta}\,|j\rangle}{\sqrt{2}}, \quad i, j \in \{0, 1, 2, 3\}, \quad i<j \tag{S.34}$$

with eignvalues ± 1. The other two vectors $|k \neq i, j\rangle, k \in \{0, 1, 2, 3\}$ corresponding to eignvalue 0. The expectations of these observables satisfy the relations:

$$
\begin{aligned}
\frac{1}{3}\left(\langle\Omega_0^{\otimes 3}(0,1)\rangle - \langle\Omega_{\pi/3}^{\otimes 3}(0,1)\rangle + \langle\Omega_{2\pi/3}^{\otimes 3}(0,1)\rangle\right) &= \langle 111|\rho|000\rangle + \langle 000|\rho|111\rangle \\
\frac{1}{3}\left(\langle\Omega_0^{\otimes 3}(0,2)\rangle - \langle\Omega_{\pi/3}^{\otimes 3}(0,2)\rangle + \langle\Omega_{2\pi/3}^{\otimes 3}(0,2)\rangle\right) &= \langle 222|\rho|000\rangle + \langle 000|\rho|222\rangle \\
\frac{1}{3}\left(\langle\Omega_0^{\otimes 3}(0,3)\rangle - \langle\Omega_{\pi/3}^{\otimes 3}(0,3)\rangle + \langle\Omega_{2\pi/3}^{\otimes 3}(0,3)\rangle\right) &= \langle 333|\rho|000\rangle + \langle 000|\rho|333\rangle \\
\frac{1}{3}\left(\langle\Omega_0^{\otimes 3}(1,2)\rangle - \langle\Omega_{\pi/3}^{\otimes 3}(1,2)\rangle + \langle\Omega_{2\pi/3}^{\otimes 3}(1,2)\rangle\right) &= \langle 222|\rho|111\rangle + \langle 111|\rho|222\rangle \\
\frac{1}{3}\left(\langle\Omega_0^{\otimes 3}(1,3)\rangle - \langle\Omega_{\pi/3}^{\otimes 3}(1,3)\rangle + \langle\Omega_{2\pi/3}^{\otimes 3}(1,3)\rangle\right) &= \langle 333|\rho|111\rangle + \langle 111|\rho|333\rangle \\
\frac{1}{3}\left(\langle\Omega_0^{\otimes 3}(2,3)\rangle - \langle\Omega_{\pi/3}^{\otimes 3}(2,3)\rangle + \langle\Omega_{2\pi/3}^{\otimes 3}(2,3)\rangle\right) &= \langle 333|\rho|222\rangle + \langle 222|\rho|333\rangle
\end{aligned}
\tag{S.35}
$$

Since the density matrix satisfies $\langle jjj|\rho|iii\rangle = \langle iii|\rho|jjj\rangle^*$, we have $\langle jjj|\rho|iii\rangle + \langle iii|\rho|jjj\rangle = 2\mathrm{Re}[\langle iii|\rho|jjj\rangle] = 2\mathrm{Re}[\langle jjj|\rho|iii\rangle]$. Thus, the formula for calculating the real part of the off-diagonal elements of the density matrix can be written as follows:

$$
6\mathrm{Re}[\langle iii|\rho|jjj\rangle] = \langle\Omega_0^{\otimes 3}(i,j)\rangle - \langle\Omega_{\pi/3}^{\otimes 3}(i,j)\rangle + \langle\Omega_{2\pi/3}^{\otimes 3}(i,j)\rangle \tag{S.36}
$$

where $i, j \in \{0, 1, 2, 3\}$ and $i<j$.

As an example, we present a proof by taking $i = 0, j = 1$. The matrix representation of $\Omega_\theta^{\otimes 3}(0,1)$ can be written as:

$$
\begin{aligned}
\Omega_\theta^{\otimes 3}(0,1) &= \begin{bmatrix} 0 & e^{-\mathrm{i}\theta} & 0 & 0 \\ e^{\mathrm{i}\theta} & 0 & 0 & 0 \\ 0 & 0 & 0 & 0 \\ 0 & 0 & 0 & 0 \end{bmatrix}^{\otimes 3} \\
&= \begin{bmatrix} 1 & 0 \\ 0 & 0 \end{bmatrix} \otimes \begin{bmatrix} 0 & 0 & 0 & 0 & 0 & e^{-2\mathrm{i}\theta} & 0 & 0 \\ 0 & 0 & 0 & 0 & 1 & 0 & 0 & 0 \\ 0 & 0 & 0 & 0 & 0 & 0 & 0 & 0 \\ 0 & 0 & 0 & 0 & 0 & 0 & 0 & 0 \\ 0 & 1 & 0 & 0 & 0 & 0 & 0 & 0 \\ e^{2\mathrm{i}\theta} & 0 & 0 & 0 & 0 & 0 & 0 & 0 \\ 0 & 0 & 0 & 0 & 0 & 0 & 0 & 0 \\ 0 & 0 & 0 & 0 & 0 & 0 & 0 & 0 \end{bmatrix} \otimes \begin{bmatrix} 0 & e^{-\mathrm{i}\theta} & 0 & 0 \\ e^{\mathrm{i}\theta} & 0 & 0 & 0 \\ 0 & 0 & 0 & 0 \\ 0 & 0 & 0 & 0 \end{bmatrix}
\end{aligned}
$$

Its expectation with the quantum state $\rho = \{\rho_{ij}\}$ $(i, j = 0, 1, 2, \cdots, 63)$ can be calculated by:

$$
\begin{aligned}
\langle\Omega_\theta^{\otimes 3}(0,1)\rangle =& \mathrm{Tr}[\rho\Omega_\theta^{\otimes 3}(0,1)] \\
=& e^{3\mathrm{i}\theta}\rho_{21,0} + e^{\mathrm{i}\theta}\rho_{20,1} + e^{\mathrm{i}\theta}\rho_{17,4} + e^{-\mathrm{i}\theta}\rho_{16,5} \\
&+ e^{\mathrm{i}\theta}\rho_{5,16} + e^{-\mathrm{i}\theta}\rho_{4,17} + e^{-\mathrm{i}\theta}\rho_{1,20} + e^{-3\mathrm{i}\theta}\rho_{0,21}
\end{aligned}
\tag{S.37}
$$

Substituting it into the right hand side of Eq. (S.36) for the case $i=0, j=1$ yields:

$$\begin{aligned}&(1-e^{3\mathrm{i}\pi/3}+e^{6\mathrm{i}\pi/3})\rho_{21,0}+(1-e^{\mathrm{i}\pi/3}+e^{2\mathrm{i}\pi/3})\rho_{20,1}+(1-e^{\mathrm{i}\pi/3}+e^{2\mathrm{i}\pi/3})\rho_{17,4}\\&+(1-e^{-\mathrm{i}\pi/3}+e^{-2\mathrm{i}\pi/3})\rho_{16,5}+(1-e^{\mathrm{i}\pi/3}+e^{2\mathrm{i}\pi/3})\rho_{5,16}+(1-e^{-\mathrm{i}\pi/3}+e^{-2\mathrm{i}\pi/3})\rho_{4,17}\\&+(1-e^{-\mathrm{i}\pi/3}+e^{-2\mathrm{i}\pi/3})\rho_{1,20}+(1-e^{-3\mathrm{i}\pi/3}+e^{-6\mathrm{i}\pi/3})\rho_{0,21}\\&=3(\rho_{21,0}+\rho_{0,21})=6\mathrm{Re}[\langle 000|\,\rho\,|111\rangle]\end{aligned} \tag{S.38}$$

For general $i,j\in\{0,1,2,3\}; i<j$, the calculation and conclusion follow analogously.

G.2 Off-diagonal element measurement of 3-ququart W state density matrix

The three-ququart W state can be written as:

$$|\mathrm{W}_3^4\rangle=\frac{1}{\sqrt{10}}\Big(|300\rangle+|030\rangle+|003\rangle+|210\rangle+|120\rangle+|201\rangle+|102\rangle+|021\rangle+|012\rangle+|111\rangle\Big) \tag{S.39}$$

Consequently, the density matrix requires measurement of 10 diagonal elements and 45 upper off-diagonal elements. The real diagonal elemenets can be calculated from the measurement results in the computational basis, while the real part of the off-diagonal elements can be measured from a group of coherence relations similar to Eq.(S.35).

Table S2: The coherence relations for calculating the 45 off-diagonal non-zero elements of the 3-ququart W state.

Off-diagonal element	Coherence relation
$\langle 300\|\rho\|030\rangle$	$\langle\Omega_{-\theta}(0,3)\otimes\Omega_\theta(0,3)\otimes\|0\rangle\langle 0\|\rangle=2\mathrm{Re}[\langle 300\|\rho\|030\rangle]\cos 2\theta$
$\langle 300\|\rho\|003\rangle$	$\langle\Omega_{-\theta}(0,3)\otimes\|0\rangle\langle 0\|\otimes\Omega_\theta(0,3)\rangle=2\mathrm{Re}[\langle 300\|\rho\|003\rangle]\cos 2\theta$
$\langle 300\|\rho\|210\rangle$	$\langle\Omega_{-\theta}(2,3)\otimes\Omega_\theta(0,1)\otimes\|0\rangle\langle 0\|\rangle=2\mathrm{Re}[\langle 300\|\rho\|210\rangle]\cos 2\theta$
$\langle 300\|\rho\|120\rangle$	$\langle\Omega_{-\theta}(1,3)\otimes\Omega_\theta(0,2)\otimes\|0\rangle\langle 0\|\rangle=2\mathrm{Re}[\langle 300\|\rho\|120\rangle]\cos 2\theta$
$\langle 300\|\rho\|201\rangle$	$\langle\Omega_{-\theta}(2,3)\otimes\|0\rangle\langle 0\|\otimes\Omega_\theta(0,1)\rangle=2\mathrm{Re}[\langle 300\|\rho\|201\rangle]\cos 2\theta$
$\langle 300\|\rho\|102\rangle$	$\langle\Omega_{-\theta}(1,3)\otimes\|0\rangle\langle 0\|\otimes\Omega_\theta(0,2)\rangle=2\mathrm{Re}[\langle 300\|\rho\|102\rangle]\cos 2\theta$
$\langle 300\|\rho\|021\rangle$	$\langle\Omega_{-\theta}(0,3)\otimes\Omega_\theta(0,2)\otimes\Omega_\theta(0,1)\rangle=2\mathrm{Re}[\langle 300\|\rho\|021\rangle]\cos 3\theta$
$\langle 300\|\rho\|012\rangle$	$\langle\Omega_{-\theta}(0,3)\otimes\Omega_\theta(0,1)\otimes\Omega_\theta(0,2)\rangle=2\mathrm{Re}[\langle 300\|\rho\|012\rangle]\cos 3\theta$
$\langle 300\|\rho\|111\rangle$	$\langle\Omega_{-\theta}(1,3)\otimes\Omega_\theta(0,1)\otimes\Omega_\theta(0,1)\rangle=2\mathrm{Re}[\langle 300\|\rho\|111\rangle]\cos 3\theta$
$\langle 030\|\rho\|003\rangle$	$\langle\|0\rangle\langle 0\|\otimes\Omega_{-\theta}(0,3)\otimes\Omega_\theta(0,3)\rangle=2\mathrm{Re}[\langle 030\|\rho\|003\rangle]\cos 2\theta$
$\langle 030\|\rho\|210\rangle$	$\langle\Omega_\theta(0,2)\otimes\Omega_{-\theta}(1,3)\otimes\|0\rangle\langle 0\|\rangle=2\mathrm{Re}[\langle 030\|\rho\|210\rangle]\cos 2\theta$
$\langle 030\|\rho\|120\rangle$	$\langle\Omega_\theta(0,1)\otimes\Omega_{-\theta}(2,3)\otimes\|0\rangle\langle 0\|\rangle=2\mathrm{Re}[\langle 030\|\rho\|120\rangle]\cos 2\theta$
$\langle 030\|\rho\|201\rangle$	$\langle\Omega_\theta(0,2)\otimes\Omega_{-\theta}(0,3)\otimes\Omega_\theta(0,1)\rangle=2\mathrm{Re}[\langle 030\|\rho\|201\rangle]\cos 3\theta$
$\langle 030\|\rho\|102\rangle$	$\langle\Omega_\theta(0,1)\otimes\Omega_{-\theta}(0,3)\otimes\Omega_\theta(0,2)\rangle=2\mathrm{Re}[\langle 030\|\rho\|102\rangle]\cos 3\theta$
$\langle 030\|\rho\|021\rangle$	$\langle\|0\rangle\langle 0\|\otimes\Omega_{-\theta}(2,3)\otimes\Omega_\theta(0,1)\rangle=2\mathrm{Re}[\langle 030\|\rho\|021\rangle]\cos 2\theta$
$\langle 030\|\rho\|012\rangle$	$\langle\|0\rangle\langle 0\|\otimes\Omega_{-\theta}(1,3)\otimes\Omega_\theta(0,2)\rangle=2\mathrm{Re}[\langle 030\|\rho\|012\rangle]\cos 2\theta$

Continued on next page

Table S2 – continued from previous page

$\langle 030\| \rho \|111\rangle$	$\langle \Omega_{\theta}(0,1) \otimes \Omega_{-\theta}(1,3) \otimes \Omega_{\theta}(0,1)\rangle = 2\mathrm{Re}[\langle 030\| \rho \|111\rangle] \cos 3\theta$
$\langle 003\| \rho \|210\rangle$	$\langle \Omega_{\theta}(0,2) \otimes \Omega_{\theta}(0,1) \otimes \Omega_{-\theta}(0,3)\rangle = 2\mathrm{Re}[\langle 003\| \rho \|210\rangle] \cos 3\theta$
$\langle 003\| \rho \|120\rangle$	$\langle \Omega_{\theta}(0,1) \otimes \Omega_{\theta}(0,2) \otimes \Omega_{-\theta}(0,3)\rangle = 2\mathrm{Re}[\langle 003\| \rho \|120\rangle] \cos 3\theta$
$\langle 003\| \rho \|201\rangle$	$\langle \Omega_{\theta}(0,2) \otimes \|0\rangle \langle 0\| \otimes \Omega_{-\theta}(1,3)\rangle = 2\mathrm{Re}[\langle 003\| \rho \|201\rangle] \cos 2\theta$
$\langle 003\| \rho \|102\rangle$	$\langle \Omega_{\theta}(0,1) \otimes \|0\rangle \langle 0\| \otimes \Omega_{-\theta}(2,3)\rangle = 2\mathrm{Re}[\langle 003\| \rho \|102\rangle] \cos 2\theta$
$\langle 003\| \rho \|021\rangle$	$\langle \|0\rangle \langle 0\| \otimes \Omega_{\theta}(0,2) \otimes \Omega_{-\theta}(1,3)\rangle = 2\mathrm{Re}[\langle 003\| \rho \|021\rangle] \cos 2\theta$
$\langle 003\| \rho \|012\rangle$	$\langle \|0\rangle \langle 0\| \otimes \Omega_{\theta}(0,1) \otimes \Omega_{-\theta}(2,3)\rangle = 2\mathrm{Re}[\langle 003\| \rho \|012\rangle] \cos 2\theta$
$\langle 003\| \rho \|111\rangle$	$\langle \Omega_{\theta}(0,1) \otimes \Omega_{\theta}(0,1) \otimes \Omega_{-\theta}(1,3)\rangle = 2\mathrm{Re}[\langle 003\| \rho \|111\rangle] \cos 3\theta$
$\langle 210\| \rho \|120\rangle$	$\langle \Omega_{-\theta}(1,2) \otimes \Omega_{\theta}(1,2) \otimes \|0\rangle \langle 0\|\rangle = 2\mathrm{Re}[\langle 210\| \rho \|120\rangle] \cos 2\theta$
$\langle 210\| \rho \|201\rangle$	$\langle \|2\rangle \langle 2\| \otimes \Omega_{-\theta}(0,1) \otimes \Omega_{\theta}(0,1)\rangle = 2\mathrm{Re}[\langle 210\| \rho \|201\rangle] \cos 2\theta$
$\langle 210\| \rho \|102\rangle$	$\langle \Omega_{-\theta}(1,2) \otimes \Omega_{-\theta}(0,1) \otimes \Omega_{\theta}(0,2)\rangle = 2\mathrm{Re}[\langle 210\| \rho \|102\rangle] \cos 3\theta$
$\langle 210\| \rho \|021\rangle$	$\langle \Omega_{-\theta}(0,2) \otimes \Omega_{\theta}(1,2) \otimes \Omega_{\theta}(0,1)\rangle = 2\mathrm{Re}[\langle 210\| \rho \|021\rangle] \cos 3\theta$
$\langle 210\| \rho \|012\rangle$	$\langle \Omega_{-\theta}(0,2) \otimes \|1\rangle \langle 1\| \otimes \Omega_{\theta}(0,2)\rangle = 2\mathrm{Re}[\langle 210\| \rho \|012\rangle] \cos 2\theta$
$\langle 210\| \rho \|111\rangle$	$\langle \Omega_{-\theta}(1,2) \otimes \|1\rangle \langle 1\| \otimes \Omega_{\theta}(0,1)\rangle = 2\mathrm{Re}[\langle 210\| \rho \|111\rangle] \cos 2\theta$
$\langle 120\| \rho \|201\rangle$	$\langle \Omega_{\theta}(1,2) \otimes \Omega_{-\theta}(0,2) \otimes \Omega_{\theta}(0,1)\rangle = 2\mathrm{Re}[\langle 120\| \rho \|201\rangle] \cos 3\theta$
$\langle 120\| \rho \|102\rangle$	$\langle \|1\rangle \langle 1\| \otimes \Omega_{-\theta}(0,2) \otimes \Omega_{\theta}(0,2)\rangle = 2\mathrm{Re}[\langle 120\| \rho \|102\rangle] \cos 2\theta$
$\langle 120\| \rho \|021\rangle$	$\langle \Omega_{-\theta}(0,1) \otimes \|2\rangle \langle 2\| \otimes \Omega_{\theta}(0,1)\rangle = 2\mathrm{Re}[\langle 120\| \rho \|021\rangle] \cos 2\theta$
$\langle 120\| \rho \|012\rangle$	$\langle \Omega_{-\theta}(0,1) \otimes \Omega_{-\theta}(1,2) \otimes \Omega_{\theta}(0,2)\rangle = 2\mathrm{Re}[\langle 120\| \rho \|012\rangle] \cos 3\theta$
$\langle 120\| \rho \|111\rangle$	$\langle \|1\rangle \langle 1\| \otimes \Omega_{-\theta}(1,2) \otimes \Omega_{\theta}(0,1)\rangle = 2\mathrm{Re}[\langle 120\| \rho \|111\rangle] \cos 2\theta$
$\langle 201\| \rho \|102\rangle$	$\langle \Omega_{-\theta}(1,2) \otimes \|0\rangle \langle 0\| \otimes \Omega_{\theta}(1,2)\rangle = 2\mathrm{Re}[\langle 201\| \rho \|102\rangle] \cos 2\theta$
$\langle 201\| \rho \|021\rangle$	$\langle \Omega_{-\theta}(0,2) \otimes \Omega_{\theta}(0,2) \otimes \|1\rangle \langle 1\|\rangle = 2\mathrm{Re}[\langle 201\| \rho \|021\rangle] \cos 2\theta$
$\langle 201\| \rho \|012\rangle$	$\langle \Omega_{-\theta}(0,2) \otimes \Omega_{\theta}(0,1) \otimes \Omega_{\theta}(1,2)\rangle = 2\mathrm{Re}[\langle 201\| \rho \|012\rangle] \cos 3\theta$
$\langle 201\| \rho \|111\rangle$	$\langle \Omega_{-\theta}(1,2) \otimes \Omega_{\theta}(0,1) \otimes \|1\rangle \langle 1\|\rangle = 2\mathrm{Re}[\langle 201\| \rho \|111\rangle] \cos 2\theta$
$\langle 102\| \rho \|021\rangle$	$\langle \Omega_{-\theta}(0,1) \otimes \Omega_{\theta}(0,2) \otimes \Omega_{-\theta}(1,2)\rangle = 2\mathrm{Re}[\langle 102\| \rho \|021\rangle] \cos 3\theta$
$\langle 102\| \rho \|012\rangle$	$\langle \Omega_{-\theta}(0,1) \otimes \Omega_{\theta}(0,1) \otimes \|2\rangle \langle 2\|\rangle = 2\mathrm{Re}[\langle 102\| \rho \|012\rangle] \cos 2\theta$
$\langle 102\| \rho \|111\rangle$	$\langle \|1\rangle \langle 1\| \otimes \Omega_{\theta}(0,1) \otimes \Omega_{-\theta}(1,2)\rangle = 2\mathrm{Re}[\langle 102\| \rho \|111\rangle] \cos 2\theta$
$\langle 021\| \rho \|012\rangle$	$\langle \|0\rangle \langle 0\| \otimes \Omega_{-\theta}(1,2) \otimes \Omega_{\theta}(1,2)\rangle = 2\mathrm{Re}[\langle 021\| \rho \|012\rangle] \cos 2\theta$
$\langle 021\| \rho \|111\rangle$	$\langle \Omega_{\theta}(0,1) \otimes \Omega_{-\theta}(1,2) \otimes \|1\rangle \langle 1\|\rangle = 2\mathrm{Re}[\langle 021\| \rho \|111\rangle] \cos 2\theta$
$\langle 012\| \rho \|111\rangle$	$\langle \Omega_{\theta}(0,1) \otimes \|1\rangle \langle 1\| \otimes \Omega_{-\theta}(1,2)\rangle = 2\mathrm{Re}[\langle 012\| \rho \|111\rangle] \cos 2\theta$

Table S2 shows the coherence relations for the 45 upper off-diagonal elements. For the coherence ralations containing factor $\cos 2\theta$ like

$$\langle \Omega_{-\theta}(0,3) \otimes \Omega_{\theta}(0,3) \otimes |0\rangle \langle 0|\rangle = 2\mathrm{Re}[\langle 300| \rho |030\rangle] \cos 2\theta \tag{S.40}$$

the corresponding real part of the off-diagonal elements can be measured by setting $\theta = 0, \pi/2$ and performing positive-negative summation:

$$\begin{aligned}\mathrm{Re}[\langle 300| \rho |030\rangle] = \frac{1}{4}\Big(& \langle \Omega_{-0}(0,3) \otimes \Omega_{0}(0,3) \otimes |0\rangle \langle 0|\rangle \\ & - \langle \Omega_{-\pi/2}(0,3) \otimes \Omega_{\pi/2}(0,3) \otimes |0\rangle \langle 0|\rangle \Big)\end{aligned} \tag{S.41}$$

For the coherence relations containing factor $\cos 3\theta$ like

$$\langle \Omega_{-\theta}(0,3) \otimes \Omega_{\theta}(0,2) \otimes \Omega_{\theta}(0,1) \rangle = 2\mathrm{Re}[\langle 300| \rho |021\rangle] \cos 3\theta \tag{S.42}$$

the corresponding real part of the off-diagonal elements can be measured by setting $\theta = 0, \pi/3, 2\pi/3$ and performing positive-negative-positive summation:

$$\begin{aligned} \mathrm{Re}[\langle 300| \rho |021\rangle] = \frac{1}{6}\Big(& \langle \Omega_{-0}(0,3) \otimes \Omega_{0}(0,2) \otimes \Omega_{0}(0,1) \rangle \\ & - \langle \Omega_{-\pi/3}(0,3) \otimes \Omega_{\pi/3}(0,2) \otimes \Omega_{\pi/3}(0,1) \rangle \\ & + \langle \Omega_{-2\pi/3}(0,3) \otimes \Omega_{2\pi/3}(0,2) \otimes \Omega_{2\pi/3}(0,1) \rangle \Big) \end{aligned} \tag{S.43}$$

We present proofs for Eq.(S.41) and Eq.(S.43). Proofs for other coherence relations are similar.

For Eq.(S.41), the matrix representation of $\Omega_{-\theta}(0,3) \otimes \Omega_{\theta}(0,3) \otimes |0\rangle\langle 0|$ can be written as:

$$\begin{aligned} & \Omega_{-\theta}(0,3) \otimes \Omega_{\theta}(0,3) \otimes |0\rangle\langle 0| \\ = & \begin{bmatrix} 0 & 0 & 0 & e^{\mathrm{i}\theta} \\ 0 & 0 & 0 & 0 \\ 0 & 0 & 0 & 0 \\ e^{-\mathrm{i}\theta} & 0 & 0 & 0 \end{bmatrix} \otimes \begin{bmatrix} 0 & 0 & 0 & e^{-\mathrm{i}\theta} \\ 0 & 0 & 0 & 0 \\ 0 & 0 & 0 & 0 \\ e^{\mathrm{i}\theta} & 0 & 0 & 0 \end{bmatrix} \otimes \begin{bmatrix} 1 & 0 & 0 & 0 \\ 0 & 0 & 0 & 0 \\ 0 & 0 & 0 & 0 \\ 0 & 0 & 0 & 0 \end{bmatrix} \\ = & \begin{bmatrix} 0 & 0 & 0 & 0 & \cdots & 0 & 0 & 0 & 1 \\ 0 & 0 & 0 & 0 & \cdots & 0 & 0 & 0 & 0 \\ 0 & 0 & 0 & 0 & \cdots & 0 & 0 & 0 & 0 \\ 0 & 0 & 0 & 0 & \cdots & e^{2\mathrm{i}\theta} & 0 & 0 & 0 \\ \vdots & \vdots & \vdots & \vdots & & \vdots & \vdots & \vdots & \vdots \\ 0 & 0 & 0 & e^{-2\mathrm{i}\theta} & \cdots & 0 & 0 & 0 & 0 \\ 0 & 0 & 0 & 0 & \cdots & 0 & 0 & 0 & 0 \\ 0 & 0 & 0 & 0 & \cdots & 0 & 0 & 0 & 0 \\ 1 & 0 & 0 & 0 & \cdots & 0 & 0 & 0 & 0 \end{bmatrix} \otimes \begin{bmatrix} 1 & 0 & 0 & 0 \\ 0 & 0 & 0 & 0 \\ 0 & 0 & 0 & 0 \\ 0 & 0 & 0 & 0 \end{bmatrix} \end{aligned}$$

Its expectation with the quantum state $\rho = \{\rho_{ij}\}$ $(i, j = 0, 1, 2, \cdots, 63)$ can be calculated by:

$$\begin{aligned} & \mathrm{Tr}[\rho \Omega_{-\theta}(0,3) \otimes \Omega_{\theta}(0,3) \otimes |0\rangle\langle 0|] \\ = & \rho_{60,0} + e^{-2\mathrm{i}\theta} \rho_{48,12} + e^{2\mathrm{i}\theta} \rho_{12,48} + \rho_{0,60} \end{aligned} \tag{S.44}$$

Substituting it into the right hand side of Eq.(S.41) yields:

$$\begin{aligned} & \frac{1}{4}\left[\rho_{60,0}(1-1) + \rho_{48,12}(1 - e^{-\mathrm{i}\pi}) + \rho_{12,48}(1 - e^{\mathrm{i}\pi}) + \rho_{0,60}(1-1)\right] \\ = & \frac{1}{2}\left(\rho_{48,12} + \rho_{12,48}\right) = \mathrm{Re}[\langle 300| \rho |030\rangle] \end{aligned} \tag{S.45}$$

For Eq.(S.43), the matrix representation of $\Omega_{-\theta}(0,3)\otimes\Omega_\theta(0,2)\otimes\Omega_\theta(0,1)$ is:

$$\begin{aligned}
&\Omega_{-\theta}(0,3)\otimes\Omega_\theta(0,2)\otimes\Omega_\theta(0,1)\\
=&\begin{bmatrix}0&0&0&e^{\mathrm{i}\theta}\\0&0&0&0\\0&0&0&0\\e^{-\mathrm{i}\theta}&0&0&0\end{bmatrix}\otimes\begin{bmatrix}0&0&e^{-\mathrm{i}\theta}&0\\0&0&0&0\\e^{\mathrm{i}\theta}&0&0&0\\0&0&0&0\end{bmatrix}\otimes\begin{bmatrix}0&e^{-\mathrm{i}\theta}&0&0\\e^{\mathrm{i}\theta}&0&0&0\\0&0&0&0\\0&0&0&0\end{bmatrix}\\
=&\begin{bmatrix}0&0&0&0&\cdots&0&0&1&0\\0&0&0&0&\cdots&0&0&0&0\\0&0&0&0&\cdots&e^{2\mathrm{i}\theta}&0&0&0\\0&0&0&0&\cdots&0&0&0&0\\\vdots&\vdots&\vdots&\vdots&&\vdots&\vdots&\vdots&\vdots\\0&0&e^{-2\mathrm{i}\theta}&0&\cdots&0&0&0&0\\0&0&0&0&\cdots&0&0&0&0\\1&0&0&0&\cdots&0&0&0&0\\0&0&0&0&\cdots&0&0&0&0\end{bmatrix}\otimes\begin{bmatrix}0&e^{-\mathrm{i}\theta}&0&0\\e^{\mathrm{i}\theta}&0&0&0\\0&0&0&0\\0&0&0&0\end{bmatrix}
\end{aligned}\tag{S.46}$$

Its expectation with the quantum state $\rho=\{\rho_{ij}\}$ $(i,j=0,1,2,\cdots,63)$ can be calculated by:

$$\begin{aligned}
&\mathrm{Tr}[\rho\Omega_{-\theta}(0,3)\otimes\Omega_\theta(0,2)\otimes\Omega_\theta(0,1)]\\
=&e^{\mathrm{i}\theta}\rho_{57,0}+e^{-\mathrm{i}\theta}\rho_{56,1}+e^{-\mathrm{i}\theta}\rho_{49,8}+e^{-3\mathrm{i}\theta}\rho_{48,9}\\
+&e^{3\mathrm{i}\theta}\rho_{9,48}+e^{\mathrm{i}\theta}\rho_{8,49}+e^{\mathrm{i}\theta}\rho_{1,56}+e^{-\mathrm{i}\theta}\rho_{0,57}
\end{aligned}\tag{S.47}$$

Substituting it into the right hand side of Eq.(S.43) yields:

$$\begin{aligned}
&\frac{1}{6}\Big[\rho_{57,0}(1-e^{\mathrm{i}\pi/3}+e^{2\mathrm{i}\pi/3})+\rho_{56,1}(1-e^{-\mathrm{i}\pi/3}+e^{-2\mathrm{i}\pi/3})\\
&+\rho_{49,8}(1-e^{-\mathrm{i}\pi/3}+e^{-2\mathrm{i}\pi/3})+\rho_{48,9}(1-e^{-\mathrm{i}\pi}+e^{-2\mathrm{i}\pi})+\rho_{9,48}(1-e^{\mathrm{i}\pi}+e^{2\mathrm{i}\pi})\\
&+\rho_{8,49}(1-e^{\mathrm{i}\pi/3}+e^{2\mathrm{i}\pi/3})+\rho_{1,56}(1-e^{\mathrm{i}\pi/3}+e^{2\mathrm{i}\pi/3})+\rho_{0,57}(1-e^{-\mathrm{i}\pi/3}+e^{-2\mathrm{i}\pi/3})\Big]\\
&=\frac{1}{2}\left(\rho_{48,9}+\rho_{9,48}\right)=\mathrm{Re}[\langle 300|\,\rho\,|021\rangle]
\end{aligned}\tag{S.48}$$

Thus, we have proven Eqs.(S.41) and (S.43). For the remaining terms in Tab.S2, corresponding equations can be derived based on whether they involve $\cos 2\theta$ or $\cos 3\theta$ and proven analogously.
